# Synergistic Antitumor Activity of Talazoparib and Temozolomide in Malignant Rhabdoid Tumors

**DOI:** 10.3390/cancers16112041

**Published:** 2024-05-28

**Authors:** Elena Mironova, Sebastian Molinas, Vanessa Del Pozo, Abhik M. Bandyopadhyay, Zhao Lai, Dias Kurmashev, Eric L. Schneider, Daniel V. Santi, Yidong Chen, Raushan T. Kurmasheva

**Affiliations:** 1 Greehey Children’s Cancer Research Institute, University of Texas Health Science Center, San Antonio, TX 78229, USA; 2 Department of Molecular Medicine, University of Texas Health Science Center, San Antonio, TX 78229, USA; 3 Prolynx, Inc., San Francisco, CA 94107, USA; 4 Department of Population Health Sciences, University of Texas Health Science Center, San Antonio, TX 78229, USA

**Keywords:** malignant rhabdoid tumor, SMARCB1, PARP1, DNA damage and repair, pediatric cancer therapy

## Abstract

**Simple Summary:**

Mutation of the *SMARCB1* gene can cause one of the most aggressive and lethal cancers of early childhood and infancy, malignant rhabdoid tumor (MRT). Despite the standard multimodal therapy (resection, conventional chemotherapy, and radiotherapy), the outlook for young children with MRT is poor. For infants, the disease can also preclude the use of radiotherapy. Numerous experimental treatments explore epigenetic mechanisms, but the DNA damage response has not yet been extensively evaluated as a therapeutic approach for MRT. We report a new therapeutic strategy for *SMARCB1*-deficient MRTs, combining PARP1 inhibition and DNA damage induction. The observed synergy between the PEGylated PARP1 inhibitor talazoparib (PEG~TLZ) and the DNA alkylating agent temozolomide (TMZ) may lead to improved therapeutic strategies for patients with this challenging cancer. We identified a new potential biomarker of response to PEG~TLZ+TMZ, O^6^-methylguanine methyltransferase (MGMT), and uncovered dysregulated signaling pathways involved in the response. Additionally, we elucidated the pro-survival role of *SMARCB1* loss in MRT cells.

**Abstract:**

Malignant rhabdoid tumors (MRTs) are among the most aggressive and treatment-resistant malignancies affecting infants, originating in the kidney, brain, liver, and soft tissues. The 5-year event-free survival rate for these cancers is a mere 20%. In nearly all cases of MRT, the *SMARCB1* gene (occasionally *SMARCA4*)—a pivotal component of the SWI/SNF chromatin remodeling complex—is homozygously deleted, although the precise etiology of these tumors remains unknown. While young patients with localized MRT generally show improved outcomes, especially those who are older and have early-stage disease, the overall prognosis remains poor despite optimal standard treatments. This highlights the urgent need for more effective treatment strategies. We investigated the antitumor activity of a PARP1 inhibitor (talazoparib, TLZ) combined with a DNA alkylating agent (temozolomide, TMZ) in MRT xenograft models. PARP1 is a widely targeted molecule in cancer treatment and, beyond its role in DNA repair, it participates in transcriptional regulation by recruiting chromatin remodeling complexes to modulate DNA accessibility for RNA polymerases. To widen the therapeutic window of the drug combination, we employed PEGylated TLZ (PEG~TLZ), which has been reported to reduce systemic toxicity through slow drug release. Remarkably, our findings indicate that five out of six MRT xenografts exhibited an objective response to PEG~TLZ+TMZ therapy. Significantly, the loss of *SMARCB1* was found to confer a protective effect, correlating with higher expression levels of DNA damage and repair proteins in *SMARCB1*-deficient MRT cells. Additionally, we identified MGMT as a potential biomarker indicative of in vivo MRT response to PEG~TLZ+TMZ therapy. Moreover, our analysis revealed alterations in signaling pathways associated with the observed antitumor efficacy. This study presents a novel and efficacious therapeutic approach for MRT, along with a promising candidate biomarker for predicting tumor response.

## 1. Introduction

Rhabdoid tumors of the kidney (RTKs) and atypical teratoid/rhabdoid tumors (AT/RTs) constitute a group of highly malignant pediatric cancers, known as malignant rhabdoid tumors (MRTs) [1,2]. Distinguished by distinct histological features, particularly the presence of rhabdoid cells, MRTs are notorious for their occurrence in various anatomical locations including the kidneys, brain, and soft tissues [1]. These tumors are further characterized based on their site of origin, with RTKs and other soft tissue rhabdoid tumors classified as extracranial MRTs, whereas AT/RTs arise in the brain and hence are termed intracranial MRTs. The incidence of MRTs is notably elevated in patients under the age of 5, with over 80% of cases observed in children below the age of 2, and the prognosis for infants is exceedingly dismal [3,4].

The standard treatment approach for patients diagnosed with AT/RT typically involves a combination of surgery, radiation therapy, and chemotherapy. Chemotherapy regimens commonly incorporate a mix of platinum agents, epipodophyllotoxins, oxazaphosphorines, vinca alkaloids, methotrexate, and anthracycline, sometimes supplemented with intrathecal agents such as methotrexate, hydrocortisone, cytarabine, mafosfamide, and/or high-dose chemotherapy with stem cell rescue [5,6,7,8,9,10]. Historically, RTKs were managed similarly to Wilms tumors in National Wilms’ Tumor Study trials, utilizing regimens comprising vincristine, dactinomycin, and doxorubicin, with or without cyclophosphamide. While the prognosis for AT/RTs has shown improvement, with approximately 50% survival rates, the outlook for RTKs remains stagnant at around 20–25% [11,12]. The Children’s Oncology Group (COG) clinical trial for AT/RT (NCT00653068) implemented a treatment protocol involving surgery and two cycles of chemotherapy (cisplatin, cyclophosphamide, etoposide, vincristine, methotrexate), followed by three cycles of high-dose chemotherapy (thiotepa, carboplatin) with stem cell rescue. However, despite these treatments, the survival rate at 4 years for children diagnosed before 6 months of age is less than 10%. This underscores the critical necessity for a deeper understanding of the molecular mechanisms underlying MRT pathogenesis to facilitate the development of more effective treatment modalities.

Despite their aggressive clinical behavior, MRTs generally exhibit diploidy and genomic stability, devoid of recurrent gene amplifications. A singular defining feature of MRTs is the biallelic inactivation of the *SMARCB1* (SWI/SNF-related matrix-associated actin-dependent regulator of chromatin subfamily B member 1) gene, also known as *INI1*, *SNF5*, or *BAF47* [13,14,15,16], albeit rarely the *SMARCA4* gene [17,18,19]. The *SMARCB1* gene encodes a core component of the SWI/SNF chromatin remodeling complex, a multimeric assembly that modulates gene expression by alerting chromatin structure, thereby impacting cellular processes including cell cycle regulation, differentiation, and DNA repair [1,13,20,21,22]. Recognized as a tumor suppressor gene, the loss of *SMARCB1* is nearly universal in MRTs, rendering it a specific diagnostic marker for this disease. In physiological contexts, *SMARCB1* plays a pivotal role in developmental processes owing to its regulatory function in gene expression [23]. The deletion of *SMARCB1* has been linked to sensitivity to EZH2 inhibitors [24]. However, preclinical evaluations demonstrated only moderate efficacy of the single agent EZH2 inhibitor EPZ011989 in MRT xenografts, achieving prolonged time to event but no tumor regression [25]. Although the phase I/II clinical trial of tazemetostat in adult patients with soft tissue sarcoma (NCT02601950) yielded promising results for those with advanced epithelioid sarcoma, there persists a pressing need for an effective therapy for MRT patients. Hence, investigating SMARCB1 in the context of DNA damage and repair in MRTs is not only imperative for elucidating the pathogenesis of these aggressive tumors but also holds potential for the development of more efficacious therapeutic strategies. 

PARP1 (poly(ADP) ribose polymerase) is an ADP ribosylating enzyme that becomes activated upon binding to DNA single- and double-strand breaks [26,27,28,29]. Its role involves inhibiting transcription and recruiting the polycomb complex to transform chromatin into a transcriptionally repressed state [30,31,32,33]. Recently, the deficiency of ARID1A, a subunit of the SWI/SNF family, has been demonstrated to sensitize cancer cells to PARP1 inhibitors both in vitro and in vivo, rendering PARP1 an appealing target for MRT therapy [34,35,36]. Our strategy aimed to exploit the genetic vulnerability of *SMARCB1* through the combined inhibition of DNA repair using talazoparib (TLZ) and induction of DNA damage with temozolomide (TMZ). However, a potential concern arises regarding systemic toxicity previously observed with this drug combination in Ewing sarcoma [37,38]. To address this, we utilized PEGylated TLZ (PEG~TLZ), a nano-formulated PARP1 inhibitor known for its slow release characteristics, thereby minimizing systemic drug exposure [39,40].

Here, we report a novel therapeutic approach for MRTs that demonstrates high efficacy while minimizing systemic toxicity, achieved through the combination of PEG~TLZ and TMZ. Notably, we observed a reverse correlation between the expression of O^6^-methylguanine methyltransferase (MGMT)—a protein responsible for repairing methylated guanine and thus protecting chromosomes from alkylating agents like TMZ [41]—and the tumor response to PEG~TLZ+TMZ. Furthermore, inhibiting MGMT sensitized MRT cells further to the drug combination, underscoring its potential as a therapeutic target. Our findings also reveal that the deletion of *SMARCB1* is associated with the improved survival of MRT cells and increased expression of DNA damage and repair (DDR) proteins. Through transcriptomic analysis, we delineated dysregulated signaling pathways, including EGFR, Ephrin, and FOXP1, in xenografts that respond favorably to treatment.

## 2. Materials and Methods

### 2.1. Cell Lines

Rh-18, BT-12, and BT-16 cell lines were provided by Dr. Peter Houghton (University of Texas Health at San Antonio, San Antonio, TX, USA). Rh-18 was originally classified as embryonal rhabdomyosarcoma but has since been re-classified as MRT based on extensive molecular characterization and the discovery of the loss of the *SMARCB1* gene and protein expression (Supplemental Figure S5A and Figure 7A). The G401 isogenic pair (gift from Dr. Charles Roberts, St. Jude Children’s Hospital, Memphis, TN, USA) includes a control GFP-tagged G401^SMARCB1−/−^ and G401^SMARCB1+/+^ cell lines [42]. The G401^SMARCB1−/−^ cells are GFP-tagged, and fluorescence is induced by doxycycline (DOX; 1 μg/mL) treatment. *SMARCB1* expression in the G401^SMARCB1+/+^ cell line is induced by DOX. Both cell lines were cultured in McCoy’s 5A medium (16600/082, GIBCO) with 10% of heat-inactivated FBS (Millipore Sigma, F4135, Burlington, MA, USA). The EW-8 (Ewing sarcoma) cell line was cultured in RPMI-1640 medium (Corning, MT10013CV, Corning, NY, USA) with 10% heat-inactivated FBS (Millipore Sigma, F4135). Cells were maintained at 37 °C in a humidified atmosphere with 5% CO_2_. 

### 2.2. Cell Proliferation and Cytotoxicity Assay

Cell proliferation was analyzed with the IncuCyte^®^ S3 Live-Cell Analysis System. Cells were plated in 96-well plates at 3000 cells/well and treated with 1 μg/mL DOX for 24 h. TLZ (0–1000 nM), TMZ (0–3000 μM), O^6^-benzylguanine (O^6^BG) (25 μM), or combinations (O^6^BG (25 μM) + TLZ (IC_50_) + TMZ (0–3000 μM)) were added to wells 24 h after cell seeding and incubated for 72 h. The IncuCyte^®^ software (Sx5 G/O/NIR 2022A rev.1) was set up to image the cells every 6 h and was used to generate proliferation curves.

The Alamar Blue^®^ assay was used to assess G401^SMARCB1−/−^ and G401^SMARCB1+/+^ cell viability (BUF012B, BioRad, Hercules, CA, USA). Cells were plated in 96-well plates at 3000 cells/well. TLZ (MedChemExpress, HY-16106, Shanghai, China), TMZ (MedChemExpress, HY-17364/CS-0943), and the TLZ+TMZ combination at the previously mentioned concentrations were added to wells 24 h after cell seeding and incubated for 72 h, after which Alamar Blue (10% of culture volume) was added to each well for 4 h, and fluorescence was measured at 590 nm (excitation 530 nm) on a Clariostar microplate reader (BMG, Labtech, Ortenberg, Germany). Wells with untreated cells containing DMEM (sh30285, Hyclone, Cytiva, South Logan, UT, USA), 10% FBS (Millipore Sigma), and 10% *v*/*v* Alamar Blue were used as controls. Wells with culture medium without cells containing 10% *v*/*v* Alamar Blue were used to blank-correct all data. Fluorescence was recorded on a Spectra Max plate reader, using the Alamar Blue protocol provided by Softmax Pro 7.1 Software. Concentration–response curves were plotted, and IC_50_ values were interpolated from nonlinear regressions using Prism 9 (GraphPad software, La Jolla, CA, USA).

### 2.3. Clonogenic Potential Analysis

For the colony formation assay, G401^SMARCB1−/−^ and G401^SMARCB1+/+^ cells were seeded (1000/well) in a 6-well plate, pre-treated with DOX for 24 h, and then exposed to TLZ (0–50 nM), TMZ (0–50 μM), or TLZ+TMZ (TMZ range 0–50 μM, TLZ range 0–50 nM) for 9 days. Thereafter, cells were washed twice with PBS, cells were fixed with 4% paraformaldehyde, and stained with 0.5% crystal violet. Stained colonies were digitally scanned (CanoScan 5600F, Canon, Ota City, Tokyo, Japan), and colonies (with more than 50 cells each) were counted and analyzed using ImageJ software (v 1.63). At least 3 independent experiments were performed.

### 2.4. Flow Cytometry Cell Cycle Assay

To determine the effect of TLZ, TMZ, and their combination on cell cycle progression, G401^SMARCB1−/−^ and G401^SMARCB1+/+^ isogenic cell lines were pre-treated with DOX (1 μg/mL, 24 h) and cultured in the presence or absence of TMZ (IC_50_ = 395 μM (G401^SMARCB1−/−^) and IC_50_ = 446 μM (G401^SMARCB1+/+^)), TLZ (IC_50_ = 176 nM (G401^SMARCB1−/−^) and IC_50_ = 95 nM (G401^SMARCB1+/+^)), or a combination in 5% CO_2_ at 37 °C. After 72 h, the cells were detached by acutase (SigmaAldrich, St. Louis, MI, USA), fixed with ice-cold 100% ethanol, and stained with Fx Cycle PI/RNase staining solution (Invitrogen, F10797, Waltham, MA, USA). The percentage of cells at different cell cycle stages (G0/G1, G2/M, and S phases) was determined based on DNA content by BD LSRFortessa X-20 and analyzed in FlowJo 10.9.0 Software (Flow cytometer, BD Biosciences, Franklin Lakes, NJ, USA).

### 2.5. Apoptotic Cell Death Analysis

To determine the effect of TLZ, TMZ, or a combination on apoptosis, G401^SMARCB1−/−^ and G401^SMARCB1+/+^ cells were pre-treated with DOX (1 μg/mL, 24 h) and cultured in the absence or presence of TMZ (IC_50_ = 395 μM (G401^SMARCB1−/−^) and IC_50_ = 446 μM (G401^SMARCB1+/+^)), TLZ (IC_50_ = 176 nM (G401^SMARCB1−/−^) and IC_50_ = 95 nM (G401^SMARCB1+/+^)), or a combination. After 72 h, apoptotic cell death was analyzed by staining with FLUO-conjugated IncuCyte Caspase 3/7 dye (Sartorius, #4704, Göttingen, Germany) according to the manufacturer’s instructions. Image scheduling, collection, and analysis were conducted with the IncuCyte S3 Live-Cell Analysis System and software (Sartorius, Bohemia, NY, USA). Treated cells were imaged hourly for 72 h. At each timepoint, 4 images were taken per well in both brightfield and RED channels. Images were analyzed for the number of red objects per well and plotted by Prism 9 software.

### 2.6. Confocal Imaging of DNA Damage Response Proteins

Cells were grown on square cover glasses and fixed with 4% paraformaldehyde (Santa Cruz Biotechnology, 281692, Dallas, TX, USA) at room temperature and washed three times with PBS. Slides were blocked with 5% BSA (Fisher Scientific, 9703-100, Hampton, NH, USA) and 0.3% Triton X-100 in PBS. Slides were incubated with primary antibodies 53BP1 (Cell Signaling, 4937, Danvers, DA, USA) or γ-H2A.X (Cell Signaling, 9718), diluted in 1% bovine serum albumin and 0.3% Triton X-100 in PBS, overnight at 4 °C in a humidified chamber. Sections were incubated with Alexafluor-647-conjugated secondary antibodies (Abcam, 150115, Cambridge, UK) diluted in 1% bovine serum albumin and 0.3% Triton X-100 in PBS. Slides were mounted with Prolong Gold Antifade Reagent with DAPI (Cell Signaling, 8961) and 0.17 mm thick cover glass. Images were captured with an Olympus FLUOVIEW FV3000 laser-scanning confocal microscope. Nuclear foci were quantified in Fiji, using a Foci-analyzer macro (https://github.com/BioImaging-NKI/Foci-analyzer (accessed on 27 March 2024)), as described [43]. Briefly, nuclei DAPI signals were thresholded, and a watershed operation was used to separate touching nuclei. A Difference-of-Gaussians filter was used to subtract the background after maximum intensity z-projection. Areas exceeding a set threshold were identified as foci candidates, and their maxima were used as seeds for MorpholibJ’s marker-controlled watershed segmentation using the CLIJ2/CLIJX toolbox. Subsequently, very small foci were excluded from the analysis by size filtering.

### 2.7. Protein Extraction and Western Blotting

Cells were harvested using 0.25% Trypsin–EDTA solution (25200056, Thermo Fisher Scientific), washed with ice-cold PBS, and cell pellets were lysed in radioimmunoprecipitation assay (RIPA) lysis buffer (89900, Thermo Fisher Scientific) supplemented with 1% protease and phosphatase inhibitor cocktail (A32961, Thermo Fisher Scientific). Protein lysates were incubated on ice for 10 min and then centrifuged at 14,000 rpm for 10 min at 4 °C. Protein concentration in the supernatants was determined using the Bio-Rad BCA protein Assay kit (5000002EDU, Bio-Rad). 

Xenograft tumor tissues were ground under liquid nitrogen, and ~30–50 mg of powder was used for Western blot analysis. For protein extraction, tissue powder was lysed with ice-cold RIPA lysis buffer with protease and phosphatase inhibitors. After ultra-sonication, the lysates were centrifuged at 12,000 rcf (relative centrifugal field) for 20 min at 4 °C. Protein concentrations in the lysates were determined using the BCA protein array kit, as above. Equal amounts of protein from each sample were mixed with SDS loading buffer (NP0007, Invitrogen), separated by a 10% Bis-Tris gel (Invitrogen™ NuPAGE™, NP0302BOX), and transferred to an Immobilon^®^-FL PVDF Membrane (Millipore, IPFL00005) using an iBlot transfer system. The membranes were probed with antibodies against SMARCB1 (1:1000, Cell Signaling, 91735S), MGMT (1:1000, Cell Signaling, 58121), PARP1 (1:1000, Cell Signaling, 9542), and GAPDH (1:1000, Cell Signaling, 2118). Proteins were detected with LI-COR IRDye 680RD anti-mouse and LI-COR IRDye 800CW anti-rabbit secondary antibodies (1:20,000) in the mixture of LI-COR Odyssey^®^ Blocking Buffer (TBS), 0.2% Tween 20, and 0.01% SDS. Membranes were imaged using the LI-COR Odyssey^®^ CLx Imaging System (Lincoln, NB, USA). The intensity of each protein band was quantified using Li-COR Image Studio v5.2 software and normalized against the corresponding GAPDH signal. The normalized values were used to plot data graphs (Prism 9 GraphPad software).

### 2.8. Immunohistochemistry Analysis 

Whole tissues were fixed overnight in PBS-buffered 10% formalin at 4 °C, then transferred to 70% ethanol until processing. Tumors were embedded in paraffin, cut into 5 μm thick sections, and mounted on positively charged slides. Sections were deparaffinized with high-pH Target Retrieval Solution (Agilent, Santa Clara, CA, USA) using a PT Link pre-treatment module (Agilent) and stained for SMARCA4 protein (Santa Cruz, 17796), using a Dako Autostainer Link 48 (Agilent). Sections were counterstained with hematoxylin and visualized using an EnVision FLEX DAB+ sub-chromo dye system. Images were captured on an EasyScan digital slide scanner (Motic, Hong Kong, China) at 40× magnification.

### 2.9. DNA Damage Antibody Array 

Human DNA Damage Response Array C1 (RayBiotech, AAH-DDR-1-2, Peachtree Corners, GA, USA) was composed of a nitrocellulose membrane duplicate spot of 27 DNA damage-related proteins. The G401^SMARCB1−/−^ and G401^SMARCB1+/+^ cells (1 × 10^7^ cells/mL) were plated, induced by DOX for 24 h, and treated with TMZ (IC_50_ = 395 μM (G401^SMARCB1−/−^) and IC_50_ = 446 μM (G401^SMARCB1+/+^)), TLZ (IC_50_ = 176 nM (G401^SMARCB1−/−^) and IC_50_ = 95 nM (G401^SMARCB1+/+^)), or a combination. After 72 h of treatment, cells were harvested and lysed, and 500 μg of total protein was used for each array. Tumor tissues (Rh-18, G401, RBD1, RBD2, WT-16, and BT-29) were ground under liquid nitrogen, and ~30–50 mg of powder was used for protein extraction. Homogenized tissue was lysed with ice-cold RIPA lysis buffer with protease and phosphatase inhibitors. After ultra-sonication, the lysates were centrifuged at 12,000 rcf (relative centrifugal field) for 20 min at 4 °C. Protein concentration in the lysates was determined using the Bio-Rad BCA protein Assay kit (5000002EDU, Bio-Rad). Equal amounts of total protein (500 μg) were used for each sample (membrane). All analyses were performed according to the manufacturer’s instructions. The membranes were incubated with horseradish peroxidase-conjugated antibodies followed by chemiluminescent detection reagent. The chemiluminescent signal was detected and visualized using the ChemiDoc imaging system (Bio-Rad, Hercules, CA, USA). The intensity of each protein was quantified using ImageJ software (v 1.53) and normalized against the positive control (immunoglobulin). A negative control was used to subtract the background noise. Pixel intensities were normalized to positive control and expressed as mean pixel intensity. The normalized values were used to plot data graphs using Prism 9 software. 

### 2.10. In Vivo Efficacy Study

Rh-18, RBD1, RBD2, WT-16, BT-29, and G401 MRT xenograft models (developed at the St. Jude Children’s Hospital and the Nationwide Children’s Research Hospital (obtained from Peter Houghton)) (Table 1) were transplanted in immunocompromised *NOD.CB17-Prkdc^scid^/NCrHsd* female mice, which were maintained under barrier conditions and provided with the irradiated commercial pelleted diet and sterile acidified water in bottles ad libitum. In vivo experiments were conducted using protocols and conditions approved by the Institutional Animal Care and Use Committee (# 15015) at the University of Texas Health Science Center at San Antonio (UTHSCSA). All animal handling procedures were undertaken in the class 2 biological safety cabinet (BSL-2). The room temperature was maintained at 21–26 °C, with a relative humidity between 30 and 70% and a 14:10 day–night light cycle. Treatments with PEGylated TLZ (PEG~TLZ; provided by Prolynx, Inc., San Francisco, CA, USA), TMZ, or their combination were initiated when tumors reached at least 100 mm^3^ [44]. PEG~TLZ was administered at 10 μmol/kg (equivalent of 3.8 mg/kg, 0.76 mM of TLZ) as a single dose on day 1 by IP (the dose corresponds to 10 μmol of TLZ/kg; there are 4 TLZ molecules per PEG~TLZ conjugate); TMZ was given at 40 mg/kg daily for 5 days orally (PO) starting on day 3. Tolerability testing of PEG~TLZ ± TMZ dosing schedules in non-tumor-bearing mice was previously reported [39]. Tumor volume was determined weekly by capturing two perpendicular measurements using electronic calipers. Once a tumor reached 400% of the initial tumor volume, the study was terminated [45]. If the tumor volume exceeded 400% at the weekly examination, the tumor growth rate during the last week period was calculated, and the total number of days required to reach 400% of the initial tumor volume was estimated and recorded for the time-to-event analysis. The tumor volume values were used to graph the antitumor activity plots and calculate event-free survival (EFS) rates for Kaplan–Meier curves (Prism 9).

### 2.11. Statistical Analyses

In vitro studies. Data were statistically analyzed and presented as the mean ± SEM (standard error of the mean). Unless otherwise noted, statistical significance (*p*-value) was determined by a not-paired two-tailed Student’s *t*-test using Excel (v16.78 (23100802) and Prism (v10.2.3(347)) software. For multiple factors, we used the n-way ANOVA test, considering factor interactions, followed by Tukey’s Honest Significant Difference (HSD) method (R, http://www.R-project.org). For linear regression with scatter plots, we report the 95% confidence interval, Pearson correlation coefficient, and corresponding *p*-value using the R/ggpubr package (v0.4.0). 

In vivo studies. Paired control and treated EFS distributions were profiled by Kaplan–Meier curves. An event is defined as a quadrupling of tumor volume from treatment day 0. The exact time to event is estimated by interpolating between the measurements directly preceding and following the event, assuming log-linear growth. The response classification metrics are as follows: PD (progressive disease) when <50% tumor regression throughout study and >25% tumor growth at end of study; SD (stable disease) when <50% tumor regression throughout study and ≤25% tumor growth at end of study; PR (partial response) when ≥50% tumor regression at any point during study but measurable tumor throughout study period; CR (complete response) when disappearance of measurable tumor mass during the study period occurs up to two times consecutively or intermittently any number of times; and MCR (maintained complete response) when no measurable tumor mass for at least three consecutive readings at any time after treatment has been completed. Objective tumor response includes MCR, CR, and PR. The significance of differences in EFS between experimental groups (e.g., treated vs. control) was assessed with log-rank tests. EFS distributions were summarized graphically with Kaplan–Meier plots. All statistical testing was 2-sided with a *p* < 0.05 considered to be statistically significant. 

### 2.12. RNA Sequencing and Bioinformatics Analysis

Total RNA from 6 xenograft models was isolated using the Qiagen RNeasy Mini Kit (74104, Qiagen, Hilden, Germany) and Trizol (15596026, Thermo, Waltham, MA, USA) and used for RNAseq library preparation by following the KAPA Stranded RNA-seq sample preparation guide (KAPA Biosystem, Cape Town, South Africa) according to the instructions of the manufacturer with modification: the elute-frag-prime stage was conducted at 85 °C for 2 min to allow annealing without causing fragmentation. The QC was performed by nanodrop UV measurement and by running a standard 1% agarose gel and bioanalyzer. The poly-A containing mRNA molecules were purified using poly-T oligo-attached magnetic beads; then, RNA was fragmented into small pieces and copied into first-strand cDNA using reverse transcriptase and random primers, followed by the second-strand cDNA synthesis using DNA Polymerase I and RNase H. The cDNA fragments then went through an end-repair process with the addition of a single ‘A’ base followed by the ligation of adapters. The products were then purified and enriched by PCR amplification to generate final RNA-seq libraries, which were sequenced using the Illumina NovaSeq 6000 platform at 100 bp paired-end module (Greehey Children’s Cancer Research Institute (GCCRI) Genome Sequencing Facility (GSF) at UTHSCSA). 

Upon obtaining the sequence reads (~130–200 million reads, or 75–100 million pairs), the samples were aligned to USCS hg38 human genome and mm10 mouse genome using HISAT aligner (v2.1.0) [46]. We assigned each sequence read to 4 groups, human-only, mouse-only, common, and unaligned, by using the following criteria: (1) alignment scores, (2) number of mismatched bases, and (3) length of matched segment length, from paired reads. To remove mouse RNA contamination, we extracted all reads from human-only and common groups (~78–94% reads) and then aligned to the human genome (hg38) using HISAT followed with Stringtie (v2.1.3b) to quantify gene expression levels (both in read counts and in Fragment Per Kilobase of transcript (FPKM)) for all RefSeq genes [46]. Differential analyses were performed using DESeq v1.38.0 (R/Bioconductor) for ‘good responders’ vs. ‘poor responders’ groups or ‘relapsed’ vs. ‘primary tumor’ groups [47]. Up- and downregulated genes were determined by the following criteria: (1) absolute log2 fold change > 1, (2) average FPKM > 1, and (3) multiple-test-adjusted (Benjamini–Hochberg) *p* value < 0.05. Using the differential expression fold change, we also performed Gene Set Enrichment Analysis (GSEA) [48], using the stand-alone package from the Broad Institute (v4.0.3) or the implementation provided by the ClusterProfiler package v3.12.0 (R/Bioconductior) [49]. We reported expression fold change via FPKM values averaged for each comparison group and *p*-values derived from the DESeq algorithm.

## 3. Results

### 3.1. In Vitro Evaluation of the Impact of TLZ and TMZ Treatments on the Sensitization of MRT Cells

**Figure 1:**
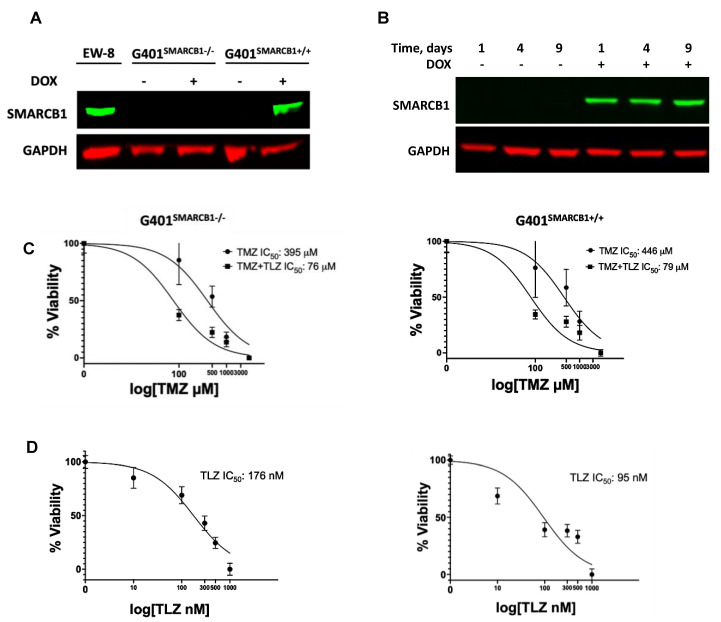
The effect of TLZ and TMZ on cell proliferation in G401 isogenic cell lines with/without *SMARCB1* re-expression. (**A**) Western blot for SMARCB1 protein in G401 isogenic cell lines. EW-8 cell line is a positive control (left lane). *SMARCB1* re-expression was induced by treatment with doxycycline (DOX) for 24 h in both *SMACRB1*-deficient G401^SMARCB1−/−^ and -proficient G401^SMARCB1+/+^ cell lines. GAPDH is a loading control. (**B**) Western blot demonstrating stable *SMARCB1* expression induction by DOX (1 μg/mL) in G401^SMARCB1+/+^ cells for up to 9 days. GAPDH is a loading control. (**C**) Alamar Blue staining in G401^SMARCB1−/−^ and G401^SMARCB1+/+^ cell lines treated with TMZ and TLZ+TMZ for 72 h. Increasing concentrations of TMZ were used alone or in combination with IC_50_ for TLZ for each cell line (shown in (**D**)). IC_50_ values were generated by Prism 9 GraphPad software. Confidence interval for this regression is provided in Supplemental Table S2. (**D**) Alamar Blue staining in G401^SMARCB1−/−^ (**left panel**) and G401^SMARCB1+/+^ (**right panel**) cell lines treated with TLZ for 72 h. IC_50_ values were generated by Prism 9 GraphPad software. Confidence interval for this regression is provided in Supplemental Table S2. Original western blots are presented in Supplementary File S1.

The in vitro growth inhibition analysis of MRT cell lines revealed the 15–110-fold TMZ potentiation by TLZ in the BT-12, BT-16, and Rh-18 cell lines, with Rh-18 cells exhibiting the most substantial sensitization (Supplemental Figure S1A–C; Supplemental Table S1). To determine whether *SMARCB1* affects MRT cell sensitivity to TLZ and TMZ, we validated the inducibility of SMARCB1 protein by DOX in the G401 isogenic lines (Figure 1A). As expected, DOX triggered SMARCB1 protein expression in the G401^SMARCB1+/+^ cell line, while it was not expressed in the control G401^SMARCB1−/−^ cells. Since most MRTs are *SMARCB1*-deficient, we aimed to compare cancer cells with ‘normal’ cells that express *SMARCB1*. We then examined cellular sensitization to TLZ, TMZ, and their combination in the isogenic cell lines using the Alamar Blue staining assay (Figure 1B). Although TLZ led to about 6-fold increase in sensitivity to TMZ, SMARCB1 expression did not further increase or decrease this sensitization (Figure 1C). Notably, re-introducing SMARCB1 had no effect on the cellular response to TMZ in either cell line, but SMARCB1-expressing cells became about twice as sensitive to TLZ (Figure 1D). These results indicate that among the MRT cell lines tested, G401 cells were the least sensitive to the TLZ+TMZ combination, while Rh-18 showed the strongest potentiation of TMZ by TLZ. Additionally, both G401 isogenic cell lines, with and without *SMARCB1* expression, were significantly more sensitive to the TLZ+TMZ combination compared to TMZ alone, indicating heightened sensitivity to PARP1 inhibition in G401^SMARCB1+/+^ cells and suggesting that the loss of *SMARCB1* could contribute to drug resistance. With this in mind, we next planned to evaluate the long-term effects of these drugs in the presence and absence of *SMARCB1*, including assessing the colony-forming potential of each cell line. 

### 3.2. Evaluation of MRT Cellular Proliferation and Clonogenic Potential in Cells Treated with TLZ and TMZ

**Figure 2:**
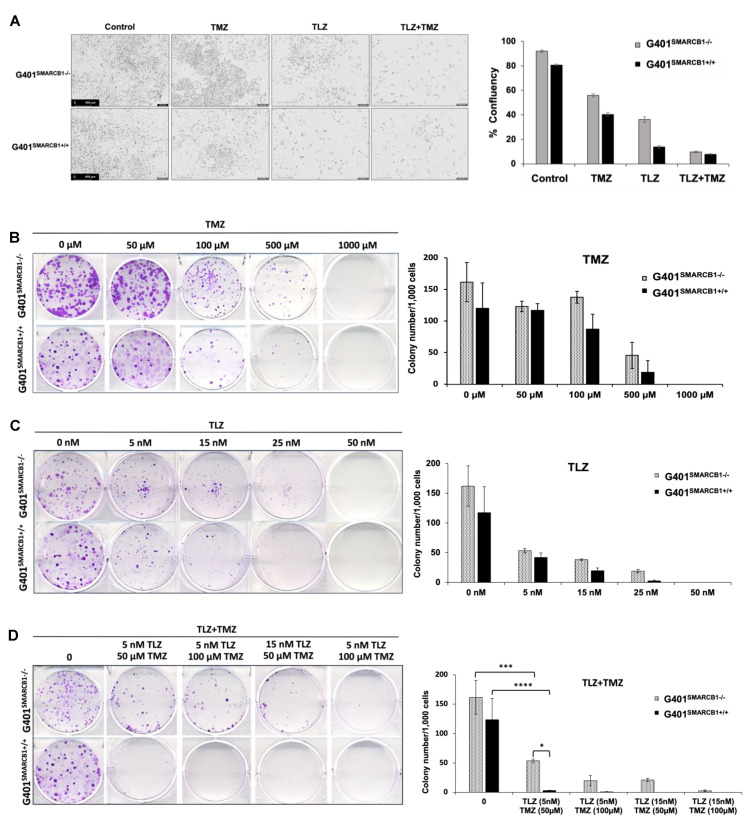
Characterization of cell proliferation and colony formation proficiency. (**A**) Effect of TLZ, TMZ, and TLZ+TMZ (at IC_50_ values) treatment on proliferation of G401^SMARCB1−/−^ and G401^SMARCB1+/+^ cells. Cells were treated with 1 μg/mL of DOX for 24 h followed by drug treatments for 3 days. ***Left panel***, representative IncuCyte images of proliferating cells. ***Right panel***, quantitation of cell surface measurements on day 4. All measurements were conducted with IncuCyte. (**B**–**D**) Clonogenic assay conducted on G401^SMARCB1−/−^ and G401^SMARCB1+/+^ cells treated for 9 days with (**B**) TMZ (0–1 mM), (**C**) TLZ (0–50 nM), or (**D**) TLZ+TMZ (0–25 nM/0–100 μM). ***Left panels***, crystal violet colony staining images. ***Right panels***, quantitation of colony numbers normalized to 1000 cells. ANOVA was performed with 2 factors (dosages and cells) and their interaction. Significant changes were reported across different dosages (*p* = 1.7 × 10^−13^) and between G401^SMARCB1−/−^ and G401^SMARCB1+/+^ cells (*p* = 0.0078). Values are shown as mean ± SEM (*n* = 3–6). Asterisk * indicates statistically significant differences of *p* ≤ 0.05, *** *p* ≤ 0.001, and **** *p* ≤ 0.0001 (*t*-test).

We initially examined the effect of TLZ, TMZ, and their combination on cell proliferation using IncuCyte, noting that each drug alone reduced cell confluence, with an even greater reduction when both TLZ and TMZ were used together in both isogenic lines (Figure 2A; Supplemental Figure S1). TLZ had a more substantial inhibitory effect on G401^SMARCB1+/+^ cells than on G401^SMARCB1−/−^ cells. To investigate the impact of TLZ combination with TMZ on the ability of MRT cells to form multicellular colonies, we performed a colony formation assay with the G401^SMARCB1−/−^ cell line and confirmed the results with G401^SMARCB1+/+^ cells. Both cell lines were exposed to various concentrations of TLZ, TMZ, and a combination of both drugs over 9 days. The *SMARCB1*-deficient G401 cells consistently formed more colonies compared to the G401^SMARCB1+/+^ cells. When normalized for colony formation, treatment with TMZ (100 μM) significantly reduced the number of colonies, but its inhibitory effect was similar for both G401^SMARCB1−/−^ and G401^SMARCB1+/+^ cells, with a reduction of 31.8% and 36.8%, respectively (Figure 2B). TLZ significantly reduced colony formation in G401^SMARCB1−/−^ at 25 nM (an 86% reduction) and in G401^SMARCB1+/+^ cells at 15 nM (a 48.7% reduction), while colony growth was completely abolished in both isogenic lines at 50 nM of TLZ (Figure 2C). The most significant effect was observed with the combination of TLZ and TMZ, which entirely inhibited colony formation in both cell lines at 50 μM TMZ combined with 15 nM TLZ (Figure 2D). However, treatments with 100 μM TMZ + 15 nM TLZ and 50 μM TMZ + 15 nM TLZ still allowed some colony formation in G401^SMARCB1−/−^ cells, while no colonies formed in G401^SMARCB1+/+^ cells under these conditions. Although G401^SMARCB1−/−^ cells could still form some colonies, the exposure of G401^SMARCB1+/+^ cells to 50 μM TMZ + 5 nM TLZ and 100 μM TMZ + 5 nM TLZ reduced colony formation by 94.4% and 96.6%, respectively (Supplemental Table S2). 

These results demonstrate that at the lowest tested concentrations of 5 nM of TLZ and 50 μM of TMZ, the combination of these drugs significantly suppresses colony formation in G401^SMARCB1−/−^ cells. Re-expression of the *SMARCB1* gene leads to slightly increased cell sensitivity to TLZ and TMZ, with this effect being more noticeable with the TLZ+TMZ combination. This suggests that *SMARCB1* deficiency in vitro might provide a protective advantage contributing to cell survival, aligning with the notion that the loss of *SMARCB1* confers drug resistance.

### 3.3. Effect of TLZ and TMZ on Cell Cycle Progression and Apoptosis in MRT Cells

**Figure 3:**
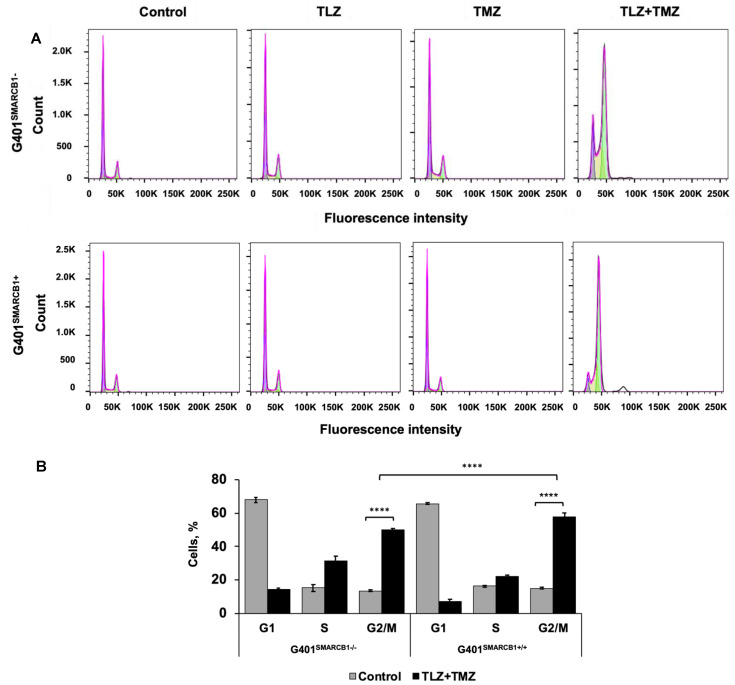
Cell cycle analysis of G401 cells treated with TLZ, TMZ, and TLZ+TMZ. (**A**) Effects of TLZ, TMZ, TLZ+TMZ, and *SMARCB1* re-expression on cell cycle distribution in two isogenic G401 cell lines were analyzed using flow cytometry. (**B**) Bar charts representing percentage of cell populations in (**A**). ANOVA was performed with 2 factors (cell cycle stages and treatment) and their interaction. Significant changes were reported across 3 cell cycle stages (*p* = 2.2 × 10^−16^), between control and TLZ+TMZ treatments (*p* = 8.6 × 10^−5^), and their interaction (*p* < 2.2 × 10^−16^). The data are presented as mean ± SEM of *n* = 6–15 replicates in 2 independent tests. Asterisks **** indicate a statistical difference of *p* ≤ 0.0001 (*t*-test). These significances were confirmed with Tukey’s HSD after ANOVA.

To understand the cell cycle events that underlie the observed growth-inhibitory effects, we evaluated the impact of the TLZ+TMZ treatment and each of these drugs individually. Both G401^SMARCB1−/−^ and G401^SMARCB1+/+^ cell lines were treated with DMSO alone as a control and IC_50_ concentrations of TLZ, TMZ, and TLZ+TMZ for 72 h (Figure 3). The samples were analyzed using a BD Fortessa Flow Cytometer (Franklin Lakes, NJ, USA), and the data were processed with FlowJo software. The cell cycle distribution between the control (DMSO), TLZ, and TMZ treatment groups showed no significant differences between G401^SMARCB1+/+^ and G401^SMARCB1−/−^ cells. However, the TLZ+TMZ combination significantly increased the cell populations in the G2/M phase in both cell lines. Moreover, the proportion of cells in the S phase was higher in SMARCB1-deficient cells compared to G401^SMARCB1+/+^ cells. 

**Figure 4:**
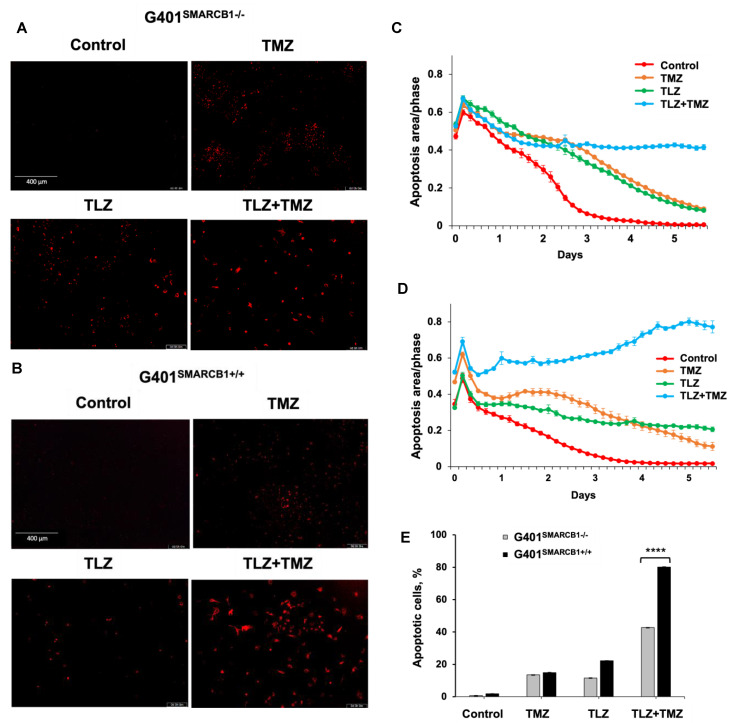
Induction of apoptosis in G401 cells by TLZ, TMZ, and TLZ+TMZ. (**A**,**B**) Representative images of G401^SMARCB1−/−^ and G401^SMARCB1+/+^ cells stained with Caspase 3/7 Red reagent following treatment with a single dose of TMZ IC_50_ (395 μM for G401^SMARCB1−/−^ and 446 μM for G401^SMARCB1+/+)^, TLZ IC_50_ (176 nM G401^SMARCB1−/−^ and 95 nM for G401^SMARCB1+/+^), and a combination of TLZ+TMZ. Detection of apoptotic cells and following analysis were conducted with IncuCyte^®^ Live Cell Imager. (**C**,**D**) Measurement of cell death in (**A**,**B**) in real time using IncuCyte over a period of 6 days. (**E**) Quantitation of apoptotic signal in (**C**,**D**) on day 5. ANOVA was performed with 2 factors (cell genotype and treatment) and their interaction. Significant changes were reported across 2 SMARCB1 genotypes (*p* < 2.2 × 10^−16^), between control, TLZ, TMZ, and TLZ+TMZ treatments (*p* < 2.2 × 10^−16^) and their interaction (*p* < 2.2 × 10^−16^). Data are presented as mean of six replicates ± SEM and compared by unpaired *t*-test; **** *p* ≤ 0.001 (*t*-test). The significant differences were confirmed between G401^SMARCB1+/+^ and G401^SMARCB1−/−^ in TLZ treatment (*p* = 8 × 10^−7^) and TLZ+TMZ treatment (*p* < 10^−7^) with Tukey’s HSD after ANOVA.

Next, we assessed the apoptotic effects of TLZ, TMZ, and the TLZ+TMZ combination in G401^SMARCB1−/−^ and G401^SMARCB1+/+^ cell lines (Figure 4). The percentage of apoptotic cells was determined by Caspase-3/7 apoptosis dye staining, following the manufacturer’s instructions, and analyzed using the IncuCyte system. After TLZ+TMZ treatment, G401^SMARCB1−/−^ cells showed a decline in the apoptotic signal over time (from 0.65 on day 1 to more than 0.42 on day 4), whereas in G401^SMARCB1+/+^ cells, the apoptotic signal increased steadily (from 0.52 on day 1 to 0.74 on day 4). This illustrates the impact of TLZ+TMZ treatment and *SMARCB1* re-expression on apoptosis induction. The effect of TLZ on apoptosis was modest in G401^SMARCB1+/+^ cells, with a slight decrease (~1.5-fold) over time (from 0.4 on day 1 to 0.24 on day 4). However, in G401^SMARCB1−/−^ cells, the apoptotic signal dropped more significantly (~3-fold), from 0.65 on day 1 to 0.22 on day 4. Thus, the number of apoptotic G401^SMARCB1+/+^ cells treated with TLZ+TMZ was significantly higher compared to G401^SMARCB1−/−^ cells. Additionally, the apoptotic effect from the TLZ+TMZ combination was more pronounced than from either drug alone. Similarly, in the TLZ-only treatment group, we observed more apoptosis in G401^SMARCB1+/+^ cells after 4 days of treatment compared to G401^SMARCB1−/−^ cells.

These findings suggest that the TLZ+TMZ combination treatment induces a significant G2/M arrest in both isogenic cell lines. G401^SMARCB1+/+^ cells exhibit a slightly stronger G2/M arrest and a reduced S-phase population compared to G401^SMARCB1−/−^ cells. Additionally, *SMARCB1*-proficient cells show about twice the rate of apoptosis after 5 days of TLZ+TMZ treatment compared to G401^SMARCB1−/−^ cells. The G401^SMARCB1+/+^ cells also exhibit increased sensitivity to TLZ alone, which aligns with the previous cytotoxicity and colony formation results. These findings further support the idea that *SMARCB1* loss confers a protective advantage to G401 cell survival.

### 3.4. Assessment of DNA Damage Induced by TLZ and TMZ in MRT Cells

**Figure 5:**
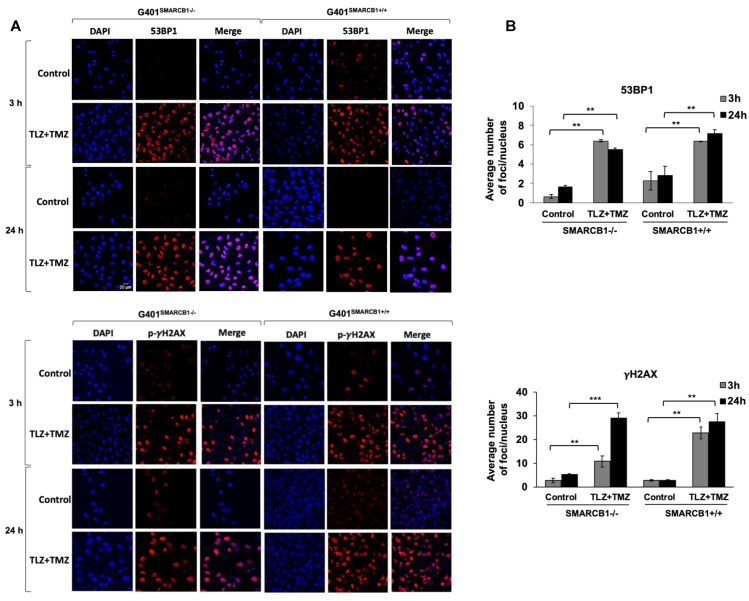
Induction of 53BP1 foci and γH2AX phosphorylation in G401 isogenic cell lines by TLZ, TMZ, and combination. (**A**) Representative images of G401^SMARCB1−/−^ and G401^SMARCB1+/+^ cells treated with TLZ (IC_50_), TMZ (IC_50_), and TLZ+TMZ for 3 and 24 h, then stained with DAPI, 53BP1, and γH2AX phospho-protein (Ser139) primary antibodies. The images were developed using an Olympus Fluoview FV3000 confocal microscope. Magnification is 60×. Scale bar is shown in the top image (20 μm). (**B**) The graphs of average number of 53BP1 foci and γH2AX phospho-protein per nucleus in G401^SMARCB1−/−^ and G401^SMARCB1+/+^ cells after treatment with TLZ, TMZ, and TLZ+TMZ (at IC_50_ concentrations) for 3, 24, and 72 h. Data are presented as mean of at least 100 nuclei ± SEM and compared by unpaired *t*-test; ** *p* ≤ 0.01; *** *p* ≤ 0.001 (*t*-test).

Our next objective was to assess the activation of DNA damage in isogenic G401 cell lines after treatment with TLZ, TMZ, and TLZ+TMZ, for 3, 24, and 72 h (Figure 5 and Supplemental Figure S2). We focused on the induction of 53BP1 expression and γH2AX phosphorylation at Ser 139, recognized as early indicators of cellular response to DNA double-strand breaks [50,51]. Combination treatment with TLZ+TMZ significantly increased the number of 53BP1 foci—by an average of 3–10 times in G401^SMARCB1−/−^ cells and 3 times in G401^SMARCB1+/+^ lines compared to control cells. It also boosted γH2AX phosphorylation (Ser 139)—by 6–8 times in G401^SMARCB1−/−^ cells and 8–10 times in G401^SMARCB1+/+^ lines compared to control cells after 3 or 24 h of treatment. The elevated levels persisted for up to 72 h (Figure 5A,B). TLZ and TMZ treatments alone also enhanced 53BP1 expression after 3 h in both G401^SMARCB1−/−^ and G401^SMARCB1+/+^ cells; however, by 72 h, the G401^SMARCB1+/+^ cells demonstrated reduced 53BP1 levels. The drop in 53BP1 foci in G401^SMARCB1+/+^ cells at later stages might be influenced by cell proliferation status, as seen in studies involving X-ray radiation treatment [52]. It is also speculated that persistent γH2AX foci result from limited availability of double-strand break repair pathways or that further replication or chromatin remodeling is needed for repair. The γH2AX phosphorylation was induced by TLZ and TMZ treatments alone at 3 h, similar to 53BP1 activation, and the expression levels did not appear to be influenced by *SMARCB1* status (Supplemental Figure S2). Overall, the number of γH2AX phosphorylation foci in both isogenic cell lines treated with TLZ+TMZ was about four times higher than that of 53BP1.

These observations suggest that the increased expression of 53BP1 and phosphorylation of γH2AX are important responses to TLZ+TMZ treatment in G401 cells. DNA damage, indicated by both 53BP1 and γH2AX, can be detected as early as 3 h after TLZ+TMZ treatment.

### 3.5. Evaluation of PEG~TLZ and TMZ Antitumor Activity in MRT Xenografts In Vivo

We next determined the efficacy of the drug combination in MRT xenograft models (Table 1). In prior studies, we used a PEGylated form of TLZ (PEG~TLZ), which helped reduce the toxicity resulting from the combination of PARP1 inhibition and alkylating DNA damage [39,40]. The tolerability of the PEG~TLZ+TMZ combination has also previously been investigated [39]. Our findings indicated that the most effective dosing schedule involved the temporal separation of individual drug administrations, allowing for a broader therapeutic index. The optimal schedule was to administer PEG~TLZ on day 1 and TMZ on day 3. 

**Figure 6:**
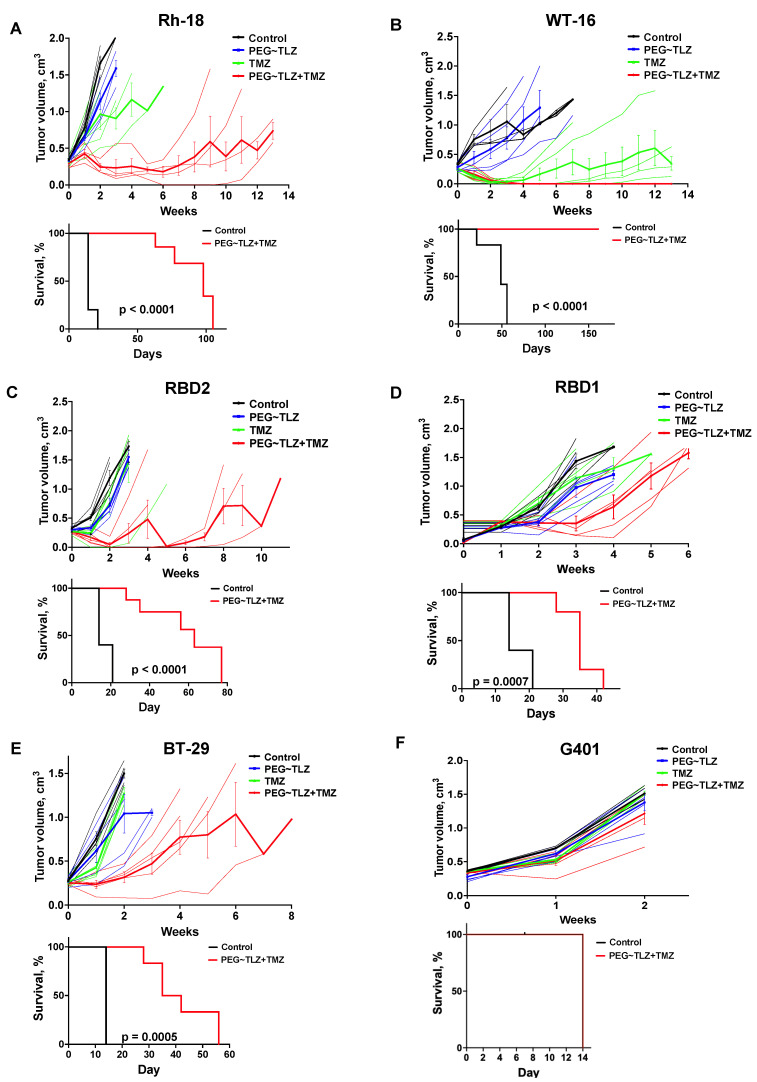
In vivo evaluation of PEG~TLZ+TMZ activity in MRT xenografts. (**A**–**F**) (***Top plots***) spider plots of individual and group mean tumor volumes for G401, Rh-28, RBD1, RBD2, BT-29, and WT-16 MRT xenograft models. Treatment groups: untreated (black), PEG~TLZ (10 μmol/kg, single dose on day 1) (blue), TMZ (40 mg/kg daily × 5 on day 3) (green), and PEG~TLZ+TMZ (red). Thinner lines represent individual mice, and bolder lines (with error bars) represent median tumor growth in each treatment group. (**A**–**F**) (***Bottom plots***) Kaplan–Meier event-free survival curves for control versus PEG~TLZ+TMZ groups. Tumor volumes were measured weekly. Error bars represent mean ± SEM. *p*-values calculated by two-sided log-rank test.

We evaluated the antitumor activity of PEG~TLZ (10 μmol/kg), TMZ (40 mg/kg), and their combination in six MRT xenograft models. The treatment schedule involved administering PEG~TLZ as a single dose on day 1 and TMZ daily for 5 days starting on day 3. The tumor volumes were measured weekly for up to 13 weeks. Objective tumor responses—defined as partial responses (PRs), complete responses (CRs), or maintained complete responses (MCRs)—were observed in five out of six MRT xenografts (WT-16, Rh-18, RBD2, RBD1, and BT-29) (Figure 6A–F; Supplemental Figures S3 and S4; Table 2). Only the G401 xenograft showed a progressive disease (PD) response. Additionally, event-free survival (EFS) was significantly longer in the PEG~TLZ+TMZ group compared to the control group, as shown in the Kaplan–Meier plots (*p* ≤ 0.0001–0.0007). Mice bearing WT-16 tumors (MCR) survived for over 161 days while on the TLZ+TMZ treatment. 

These results demonstrate the high efficacy of the PEG~TLZ+TMZ combination in MRT xenografts and its association with extended EFS.

### 3.6. Dependency of PEG~TLZ+TMZ Antitumor Activity on DDR and Other Signaling Pathways in MRT

**Figure 7:**
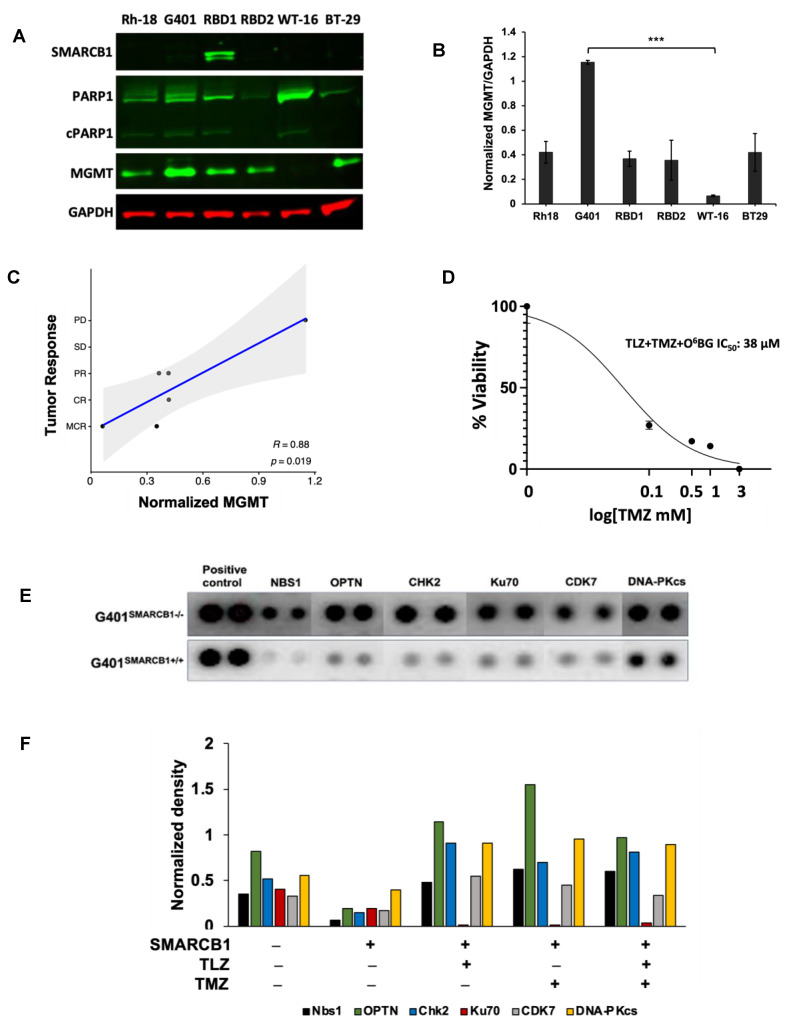
MGMT as a potential biomarker of MRT response and the therapy effect on DDR. (**A)** Western blot analysis of SMARCB1 (46 kDa), PARP1 (116 kDa)/cleaved PARP1 (cPARP1; 89 kDa), MGMT (21 kDa), and GAPDH (37 kDa) proteins. (**B**) Quantification of band intensities in (**A**) normalized to GAPDH loading control. One-way ANOVA was performed across 6 xenograft tumors. Significant change was reported with *p* = 0.0038; Tukey’s HSD further identified significant change only between G401 and WT-16 (*p* = 0.0022). Data are presented as mean ± SEM of 3 independent tests. Asterisks *** indicate a statistical difference of *p* ≤ 0.001 (*t*-test). (**C**) Inverse correlation between MGMT protein expression and the antitumor activity of PEG~TLZ+TMZ in MRT xenografts. Axis Y shows tumor response categories: 1-MCR (maintained complete response), 2-CR (complete response), 3-PR (partial response), 4-SD (stable disease), and 5-PD (progressive disease). Axis X: MGMT protein expression normalized to GAPDH. This resulted in Pearson correlation coefficient R = 0.88 with a significant *p*-value of 0.019. (**D**) G401^SMARCB1−/−^ cell potentiation to TLZ+TMZ by MGMT inhibition. Cells were treated with increasing concentrations of TMZ (0–1 mM), TLZ IC_50_ (176 nM), and O^6^BG (25 μM). Alamar Blue was added to the plates at 72 h and incubated for 4 h; fluorescence was measured at 590 nm (excitation 530 nm) on a Clariostar microplate reader. (**E**) Protein array of basal levels of DDR proteins in G401^SMARCB1−/−^ and G401^SMARCB1+/+^ cell lines. Blot was processed 24 h after treatment with DOX (1 μg/mL). The spliced fragments of the same blot are marked with thin white lines. (**F**) Plot quantitating the DDR protein expression (from the DDR protein array assay as in (**E**)) in G401^SMARCB1−/−^ and G401^SMARCB1+/+^ cells treated with TMZ (IC_50_ = 446 μM for G401^SMARCB1+/+^ and 395 μM for G401^SMARCB1−/−^), TLZ (IC_50_ = 95 nM G401^SMARCB1+/+^ and 176 nM for G401^SMARCB1−/−^), and TLZ+TMZ for 72 h. Original western blots are presented in Supplementary File S1.

The major DNA methyl adducts induced by TMZ are repaired through base excision repair mechanisms involving PARP1 and O^6^-methylguanine methyltransferase (MGMT) [53]. To understand if the expression levels of these proteins were linked to the tumor responses observed in our experiments, we examined their levels in various MRT xenograft models (Figure 7A–C). It turned out that MGMT protein expression was inversely related to the antitumor efficacy of the PEG~TLZ+TMZ combination (Table 2), indicating a new dependency for MRTs. For example, the WT-16 xenograft model, which was highly responsive to the combination treatment (MCR), did not express MGMT protein, whereas the G401 model (PD) had the highest MGMT expression. The protein expression levels were consistent with mRNA expression profiling results (Supplemental Figure S5A). Moreover, the inhibition of MGMT with O^6^-benzylguanine (O^6^BG) enhanced the potency of TLZ+TMZ by about 2-fold in G401^SMARCB1−/−^ cells, compared to treatment with TLZ+TMZ alone, suggesting that MGMT plays a significant role in the effectiveness of the combination therapy and further supporting our earlier observations (Figure 7D; Supplemental Figure S5B; Supplemental Table S2) [54]. Notably, we did not find a similar correlation for *SMARCA4* mRNA and protein expression levels. The SMARCA4 protein did not seem to predict the antitumor activity in the MRT xenograft models that we evaluated (Supplemental Figure S5A,D).

Next, we investigated how the expression of DNA damage response (DDR) proteins influenced the antitumor activity of the PEG~TLZ+TMZ combination in MRTs. Our analysis of isogenic G401 cell lines revealed that the expression of Nbs1 (Nibrin), OPTN, CHK2, CDK7, Ku70, and DNA-PKcs proteins was elevated in *SMARCB1*-deficient cells, which aligns with their increased sensitivity to DNA-damaging treatments (Figure 7E). Interestingly, treatments with TLZ, TMZ, and the combination of both drugs did not significantly affect the levels of these proteins, with the exception of Ku70, which was downregulated by TLZ (Figure 7F; Supplemental Figure S5C). These findings are in line with the observed effects of MGMT protein expression on tumor response, suggesting that the upregulation of DDR mechanisms in *SMARCB1*-deficient tumors plays a role in supporting cell survival during DNA-damaging therapies. 

The analysis of mRNA sequencing (RNAseq) data from MRT xenograft tumors indicates a significant difference in the transcriptional profiles between ‘good responders’ (those achieving CR or MCR like WT-16, Rh-18, and RBD2) and ‘poor responders’ (those achieving PR or PD such as RBD1, BT-29, and G401) (Supplemental Figure S6A). In particular, certain genes like *EGFR* (epidermal growth factor receptor), *EPHA5* (Ephrin receptor A5), and *BRINP3* (BMP/retinoic acid inducible neural specific 3) genes, known to be dysregulated in MRT [55,56,57,58,59], were highly expressed in the ‘good responders’ group but nearly undetectable in the ‘poor responders’ (Table 3, Supplemental Figure S6A,B). Conversely, *FOXP1,* a transcription factor involved in regulating stem and progenitor cell biology, had high transcript levels in the ‘poor responding’ tumors. This suggests that higher expressions of *EGFR, EPHA5,* and *BRINP3* are associated with a better response to PEG~TLZ+TMZ therapy in MRTs, while higher levels of *FOXP1* levels are linked to drug resistance. Additionally, there were genes like *CADM1*, *CCNB3*, *CDH6*, *CDH9*, *CLDN10*, *EFNB1*, *EFNB2*, *FGF3*, *FGF4*, *FGF19*, *KIT,* and *PIK3AP1* that had high expressions exclusively in the WT-16 xenograft—the model with an MCR response and a relapsed MRT model (Table 4; Supplemental Figure S6B) [60,61,62,63,64,65,66]. This suggests that transcriptional patterns could help identify which MRTs might respond better to this specific treatment regimen.

In this study, we identified MGMT as a biomarker for predicting the response to the PEG~TLZ+TMZ treatment and demonstrated that the DDR pathway is activated in *SMARCB1*-deficient MRT cells, indicating the role of *SMARCB1* loss in promoting cell survival. Additionally, we established a connection between the activation of the EGFR and EPHA5 signaling pathways, the neuronal marker BRINP3, and the antitumor activity of the PEG~TLZ+TMZ combination in MRT xenografts, suggesting potential targetable dependencies for therapy.

## 4. Discussion

We investigated the antitumor activity of TLZ, TMZ, and their combination in MRT cell lines and xenograft tumors. Our objective in conducting the in vitro studies was to uncover the relationship between the effect of the drug treatments and the *SMARCB1* re-expression on MRT cellular fitness rather than correlate cellular response to the in vivo activity of TLZ and TMZ. Although there are no matching cell lines for the xenograft tumors, the wild-type MRT cells exhibited significant TMZ sensitization when treated with TLZ. The G401 xenograft, which did not show any in vivo response to the PEG~TLZ+TMZ combination, also had the lowest in vitro potentiation of TMZ by TLZ compared to other MRT cell lines (5–6-fold vs. 15–110-fold). The loss of *SMARCB1* in G401 cells is associated with resistance to TLZ+TMZ; however, both the G401^SMARCB1−/−^ and G401^SMARCB1+/+^ cell lines are particularly sensitive to TLZ alone. Colony formation is strongly inhibited by the TLZ+TMZ combination regardless of *SMARCB1* status, with a slightly stronger effect in *SMARCB1*-proficient cells, suggesting that *SMARCB1* loss could have a protective role during extended drug treatment. The TLZ+TMZ combination also affects the cell cycle distribution, causing G2/M arrest in both isogenic cell lines, with slightly higher cell counts in the S phase and a reduced G2/M peak in G401^SMARCB1−/−^ cells. The re-expression of *SMARCB1* has been shown to arrest MRT cells in the G0/G1 phase; however, DNA damage has been shown to activate the G_2_/M checkpoint in cancer cells, which aligns with our findings [72,73,74]. Notably, TLZ alone does not cause significant cell cycle disruption despite its strong sensitization effect, suggesting other mechanisms might be involved in regulating the cellular response to TLZ. As expected with G2/M arrest, the TLZ+TMZ combination induced apoptosis in both cell lines, with slightly lower induction in *SMARCB1*-deficient cells, further supporting the cell protective role of SMARCB1 loss observed in colony formation and cell cycle analyses. Notably, the re-expression of *SMARCB1* led to a stronger inhibition of colony formation and increased apoptosis with TLZ treatment, indicating a potential dependency between PARP1 and SMARCB1. 

The in vivo study investigating the combination of PARP1 inhibition and alkylating DNA damage in MRT xenografts used a pre-defined dosing schedule for PEG~TLZ and TMZ [39]. PEG~TLZ is known for reducing toxicity and improving drug delivery due to its slow-release properties. Among the MRT xenografts studied, all but RBD1 were deficient in SMARCB1 at both protein and RNA levels, with five out of six models showing objective tumor responses (one maintained complete response (MCR), two complete responses (CRs), and two partial responses (PRs)). Although it is beyond the scope of this study, it is worth noting that the ability of PEG~TLZ to cross the blood–brain barrier has not been demonstrated, which is a consideration for AT/RT treatments in clinical settings. Given these results, SMARCB1 is unlikely to be a reliable biomarker for predicting the efficacy of PEG~TLZ+TMZ in these xenografts. Similarly, *SMARCA4* mRNA, which is highly expressed across all tested xenografts, showed no correlation with in vivo responses (Supplemental Figure S5A). In addition to PARP1, MGMT is known to repair DNA methyl adducts induced by TMZ, specifically the O^6^-methylgianune lesion [75]. An intriguing finding is that the O^6^-methylguanine methyltransferase (MGMT) protein expression inversely correlates with the antitumor activity of PEG~TLZ+TMZ. This supports a role for MGMT in the response of SMARCB1-deficient MRTs to PARP1 inhibition and DNA alkylation damage therapy. The WT-16 xenograft, which showed no basal MGMT expression, achieved MCR and prolonged survival (up to 13 weeks) after treatment with the PEG~TLZ+TMZ combination, compared to other tumors with high MGMT expression. Likewise, Rh-18 and RBD2 xenografts with CR had lower MGMT protein levels, while RBD1, BT-29, and G401 models that showed PR or PD had high MGMT expression. As previously reported, TLZ alone demonstrated low antitumor activity in rhabdomyosarcoma, Wilms tumor, neuroblastoma, most brain tumors, and osteosarcoma xenografts, despite these tumor cell lines showing significant in vitro sensitivity to TLZ (in low nanomolar range) [76]. Notably, the in vitro sensitivity of Ewing sarcoma cell lines to TLZ did not translate into in vivo activity. The two rhabdoid cell lines (BT-12 and CHLA-266) were sensitive to TLZ but within the low micromolar range (consistent with our results for BT-12 cells). These findings align with our observation of reduced colony formation in G401 cells treated with TLZ and the lack of TLZ activity in vivo. It is likely that the tumor microenvironment and genomic background contribute to the absence of TLZ activity in mice. We recently reported that PARylated MGMT has increased activity in repairing O^6^-methylguanine lesions, which is dependent on alkylating DNA damage [53]. Since resistance to TMZ can be mitigated with PARP1 inhibitors regardless of MGMT expression, mismatch repair proteins may contribute to O^6^-methylguanine repair when MGMT activity is compromised (including through PARP1 inhibition by drugs like TLZ) [77]. The relationship between MGMT protein levels and the antitumor effects of PEG~TLZ+TMZ in SMARCB1-deficient MRT models is further reinforced by a 2-fold increase in cell sensitization when using the MGMT inhibitor O^6^-benzylguanine (O^6^BG). These results indicate that MGMT expression could be a useful biomarker for predicting patient response to this combination therapy. 

Additional exploration of the DDR pathways involved in MRT response to PARP1 inhibition and DNA damage shows that re-expressing *SMARCB1* inhibits DDR-related proteins. In G401^SMARCB1−/−^ cells, compared to those with re-expressed *SMARCB1*, proteins involved in DNA repair, cell cycle arrest, or apoptosis, such as NBS1, OPTN, CHK2, Ku70, and CDK7, are upregulated [1,78,79,80,81,82,83,84,85]. This observation aligns with the enhancement of TMZ effect by PEG~TLZ in SMARCB1-deficient MRT xenografts, where DDR mechanisms are disrupted by PARP1 inhibition and alkylating DNA damage, leading to tumor cell death and combination drug efficacy. Thus, *SMARCB1* loss shows a protective effect during in vitro TLZ+TMZ treatments. In vivo studies with PEG~TLZ+TMZ demonstrate significant antitumor activity in SMARCB1-deficient MRT xenografts at doses and schedules well tolerated in mice.

Transcriptomic analysis of basal mRNA levels in MRT xenografts has revealed genes and pathways associated with the efficacy of the PEG~TLZ+TMZ treatment. An upregulation of the EGFR and Ephrin pathways, alongside a downregulation of *FOXP1,* has been found to correlate with a better in vivo response (Table 3, Supplemental Figure S6A). The upregulation of EGFR signaling has previously been shown to influence proliferation in *SMARCB1*-deficient cells, and this pathway activation was not consistently linked to *EFGR* amplification [55,57]. Similarly, *SMARCA4*-deficient MRTs, such as AT/RTs, have been noted for exhibiting high expression levels of *EPHA5,* indicating that the Ephrin signaling pathway may be tumor-promoting in *SMARCA4*-driven malignancies [56]. Additionally, the neuronal differentiation marker *BRINP3* has been linked to AT/RTs [59]. All xenograft tumors in this study express *SMARCA4* mRNA (FKPM: 22.7–100.1), suggesting an alternative mechanism responsible for *EPHA5* dependency. The *FOXP1* gene, which is involved in repressing pro-apoptotic genes, has been reported to be silenced in kidney and colon cancers, while its high expression has been associated with better prognosis in breast and lung cancers, aligning with these findings [67,68,69,70]. The WT-16 xenograft, the most responsive model, shows activation of several genes, linking PEG~TLZ+TMZ efficacy to potential biomarkers of response (Table 3 and Table 4; Supplemental Figure S6B). One of the pathways upregulated in WT-16 is FGFR signaling, which has been associated with the loss of SMARCB1 function in MRT cell lines and primary tumors [64]. WT-16 also shows increased expression of growth factors *FGF3*, *FGF4*, and *FGF19*, indicating that FGFR signaling activation may not necessarily confer resistance to PEG~TLZ+TMZ. However, the deregulated expression of receptor tyrosine kinases could play a role in drug resistance [86,87]. Further research is required to understand how these omic dependencies influence PEG~TLZ+TMZ antitumor efficacy in WT-16 and other MRT xenografts. 

## 5. Conclusions

In summary, our study demonstrates that the combination of PARP1 inhibition with DNA alkylating damage leads to reduced colony formation, enhanced G2/M cell cycle arrest, and increased apoptosis in *SMARCB1*-deficient cells. Notably, *SMARCB1* deficiency in MRT cells appears to offer a protective effect, while cell proliferation and colony formation are impacted by PARP1 inhibition regardless of *SMARCB1* status. The PEGylated form of a PARP1 inhibitor, combined with TMZ, shows significant activity in MRT models, is well tolerated in mice, and results in objective tumor responses in five out of six tested MRT xenografts. Additionally, our findings indicate that MGMT can serve as a predictive biomarker for in vivo MRT response to this treatment strategy, suggesting that modulating its expression could be a key therapeutic approach for *SMARCB1*-deficient MRTs. The upregulation of the EGFR and EPHA5 signaling pathways in MRT xenografts that respond to PEG~TLZ+TMZ therapy suggests a link between these dysregulated pathways and DDR. This points to the potential for new DDR-based therapies targeting these pathways in *SMARCB1*-deficient MRTs.

Looking ahead, future research should focus on comprehensive analyses of multi-omics data to understand the molecular basis of MRT heterogeneity and treatment responses. Identifying specific MRT subtypes with varying treatment responses and developing personalized therapeutic strategies based on distinct DDR and epigenetic profiles could ultimately lead to improved treatment outcomes for MRT patients.

## Figures and Tables

**Table 1:** MRT xenografts and demographics.

Model ID	Passage	Subtype	Age, Years	Sex	Site of Origin	Diagnosis or Relapse
BT-29	p18	AT/RT	2	M	Frontal lobe	Diagnosis
G401	p10/10	RTK	3 m	M	Kidney	Diagnosis
WT-16	p29	RTK	10 m	F	Kidney	Relapse
NCH-RBD-1	p25	Extrarenal	1	F	Lung	Diagnosis
NCH-RBD-2	p13	Extrarenal	9	F	Liver	Diagnosis
Rh18	p45	Extrarenal	2	F	Perineum	Diagnosis

AT/RT, atypical teratoid/rhabdoid tumor of the brain. RTK, rhabdoid tumor of the kidney. Passage number with double digits: 1st number corresponds to cell line, 2nd—to xenograft.

**Table 2:** EFS for PEG~TLZ+TMZ in MRT xenografts.

Tumor	Histology	Median Time to Event		Antitumor Activity
		Control (Days)	Treated (Days)	
NCH-RBD2	Extrarenal	15.2	49.5	MCR
Rh-18	Extrarenal	17.3	83.8	CR
WT-16	RTK	36.3	>161	MCR
NCH-RBD1	Extrarenal	126.6	31.7	PR
BT-29	AT/RT	10.2	34.9	PR
G401	RTK	13.3	17.8	PD

EFS, event-free survival. Event, tumor reaches 400% of its volume at the start of treatment. RTK, rhabdoid tumor of the kidney. AT/RT, atypical teratoid/rhabdoid tumor. (M)CR, (maintained) complete response. PD, progressive disease. PR, partial response.

**Table 3:** Genes uniquely over- or under-expressed in in vivo MRT ‘good’ responders.

Gene Symbol	Gene Name	Fold Change (FPKM)	Raw *p*-Value
*BRINP3* [58,59]	BMP/retinoic acid inducible neural specific 3	52.6	3.8 × 10^−6^ *
*EGFR* [55,57]	Epithelial growth factor receptor	41.8	1.4 × 10^−3^
*EPHA5* [56]	Ephrin type-A receptor 5	26.9	1.9 × 10^−4^
*FOXP1* [67,68,69,70]	Forkhead box P1	0.005	9.0 × 10^−7^ **
*MEIS3P1*	Meis homeobox 3 pseudogene 1	106.8	3.6 × 10^−4^
*VEGFC*	Vascular endothelial growth factor C	412.3	5.2 × 10^−4^

Fold change is calculated as ‘good responder’ group/’poor responder’ group. * Adjusted *p*-values (* *p* = 0.01–0.05; ** *p* = 0.001–0.01) obtained from DESeq algorithm. References shown are for reported gene role(s) in cancer. ‘Good’ responders—WT-16, Rh-18, and RBD2 xenografts. ‘Poor’ responders—RBD1, BT-29, and G401 xenografts.

**Table 4:** Genes uniquely overexpressed in WT-16 MRT xenograft.

Gene Symbol	Gene Name	Fold Change (FPKM)	Raw *p*-Value
*CADM1* [60]	Cell adhesion molecule 1	31.59	2.2 × 10^−3^ **
*CCNB3* [61]	Cyclin B3	18.06	1.1 × 10^−2^
*CDH6* [62]	Cadherin 6	173.19	8.2 × 10^−11^ ****
*CDH9*	Cadherin 9	591.03	1.2 × 10^−6^ ***
*CLDN10* [63]	Claudin 10	94.47	2.2 × 10^−3^
*EFNB1*	Ephrin B1	12.42	1.6 × 10^−3^
*EFNB2* [66]	Ephrin B2	7.41	6.7 × 10^−3^
*FGF3* [64]	Fibroblast growth factor 3	54.00	1.1 × 10^−2^
*FGF4*	Fibroblast growth factor 4	72.56	5.4 × 10^−3^
*FGF19* [65]	Fibroblast growth factor 19	2892.42	2.4 × 10^−20^ ****
*KIT*	Proto-oncogene, receptor tyrosine kinase	28.85	1.9 × 10^−2^
*PIK3AP1* [71]	Phosphoinositide-3-kinase adaptor protein 1	64.85	4.2 × 10^−5^ **

Fold change is calculated as WT-16/other xenografts. * Adjusted *p*-values (** *p* = 0.001–0.01; *** *p* = 0.0001–0.001; **** *p* < 0.0001) obtained from DESeq algorithm. References shown are for reported gene role(s) in cancer.

## Data Availability

All data are available from the corresponding author upon request.

## Supplementary Materials

The following supporting information can be downloaded at https://www.mdpi.com/article/10.3390/cancers16112041/s1: Supplemental Figure S1. Growth inhibition and proliferation analyses in MRT cell lines and the isogenic G401^SMARCB1−/−^ and G401^SMARCB1+/+^ cell lines (in support of Figure 1 and Figure 2A). Supplemental Figure S2. Representative images for remaining signal measurements of 53BP1 and H2AX (Ser 139) protein staining in G401^SMARCB1−/−^ and G401^SMARCB1+/+^ cell nuclei (in support of Figure 5). Supplemental Figure S3. Antitumor activity of PEG~TLZ, TMZ, and PEG~TLZ+TMZ in MRT xenografts (in support of Figure 5). Supplemental Figure S4. Relative tumor volume of MRT xenografts for control, PEG~TLZ, TMZ, and PEG~TLZ+TMZ treatment groups (in support of Figure 5). Supplemental Figure S5. DDR in MRT cells and xenografts (in support of Figure 6). Supplemental Figure S6. Distinctive transcriptomic profile of MRT xenograft tumors responding to PEG~TLZ+TMZ therapy (in support of Table 3 and Table 4). Supplemental Table S1. IC50 variance estimates. Supplemental Table S2. Clonogenic assay data quantification. Supplementary File S1: Original western blots.
