# Supplementary material for: Synergistic Antitumor Activity of Talazoparib and Temozolomide in Malignant Rhabdoid Tumors
Source: Cancers (Basel). 2024 May 28;16(11):2041. doi: 10.3390/cancers16112041 (PMC11171327; doi:10.3390/cancers16112041)
Supplement: Supplementary file 1 [file cancers-16-02041-s001.zip › cancers-3003518-supplementary.pdf]

SUPPLEMENTAL DATA

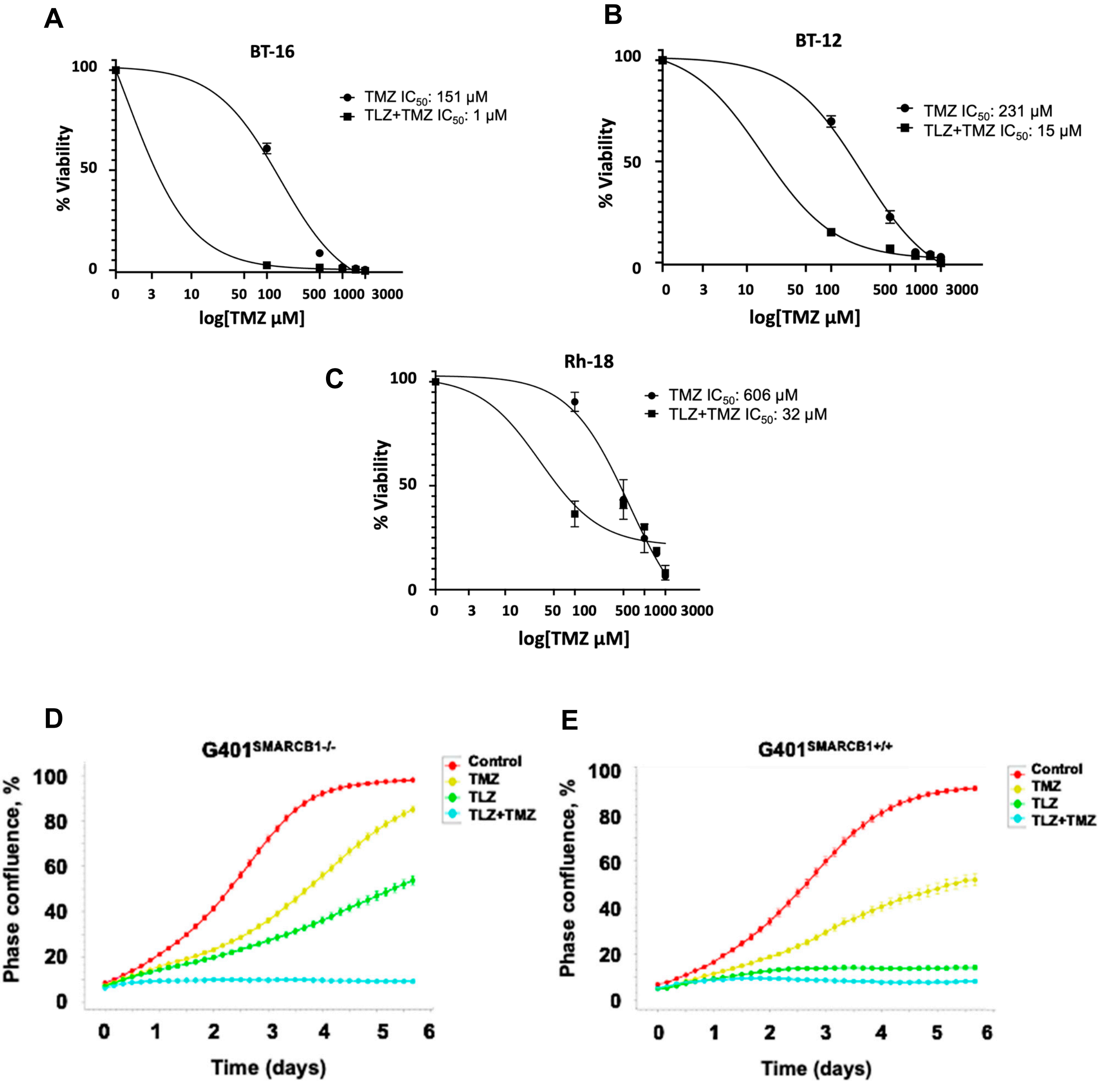

Supplemental Figure S1. Growth inhibition and proliferation analyses in MRT cell lines and the isogenic G401$^{SMARCB1-/-}$ and G401$^{SMARCB1+/+}$ cell lines (in support of Figures 1 and 2A). ***A-C,*** Growth inhibition assay for BT-12, BT-16, and Rh-18 MRT cell lines using Alamar Blue staining assay. Cells were treated with increasing TMZ concentrations (0, 3, 10, 50, 100, 300, 500, 1000, 3000 µM) and IC_{50} of TLZ for each cell line. TLZ IC_{50} in BT-16 is 10 nM, BT-12 – 964 nM, and Rh-18 – 6 nM. ***D-E,*** Effect of TLZ, TMZ, and TLZ+TMZ treatment on proliferation of G401$^{SMARCB1-/-}$ (D) and G401$^{SMARCB1+/+}$ (E) cells. Cells were treated with 1 µg/mL of DOX for 24 h followed by drug treatments (at IC_{50} values for each cell line) for 3 days. ‘0’ time point corresponds to the day of cell plating. Measurements are done by Incucyte.

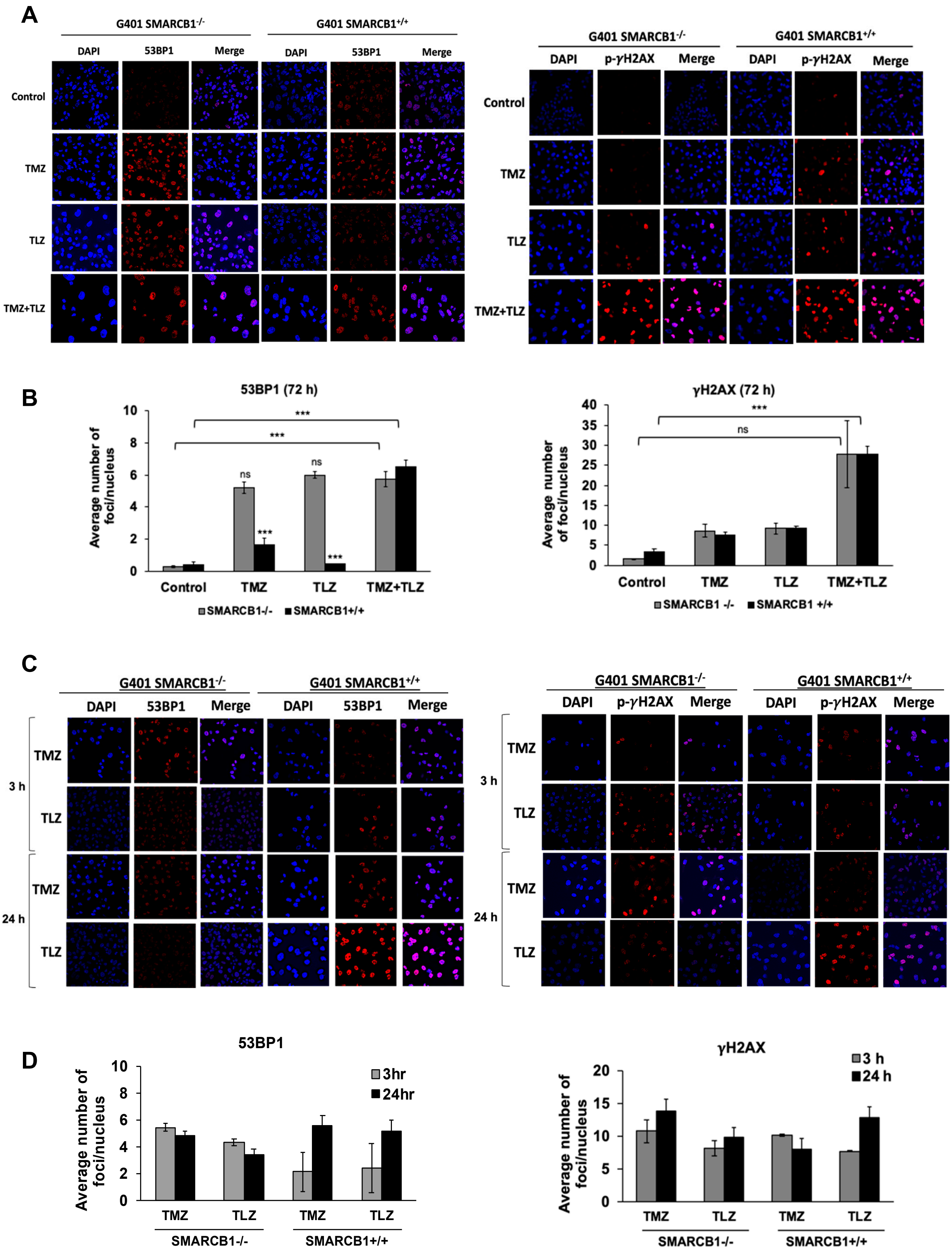

A
G401 SMARCB1-/-
G401 SMARCB1+/+
DAPI
53BP1
Merge
Control
TMZ
TLZ
TMZ+TLZ
p-γH2AX
B
53BP1 (72 h)
Average number of foci/nucleus
ns
***
Control
TMZ
TLZ
TMZ+TLZ
SMARCB1-/-
SMARCB1+/+
γH2AX (72 h)
SMARCB1 -/-
SMARCB1 +/+
C
3 h
24 h
D
53BP1
3hr
24hr
γH2AX
3 h
24 h

Supplemental Figure S2. Representative images for 53BP1 and γH2AX (Ser 139) protein staining in G401$^{SMARCB1-/-}$ and G401$^{SMARCB1+/+}$ cell nuclei (in support of Figure 5). ***A & C,*** Confocal microscope images of 53BP1 foci and γH2AX phospho-protein in cells treated with TMZ (IC_{50}), TLZ (IC_{50}), and TLZ+TMZ for 72 h (A) or images of 53BP1 foci and γH2AX phospho-protein in cells treated with TMZ (IC_{50}) and TLZ (IC_{50}) for 3 h and 24 h (C). ***B & D,*** Quantitation of fluorescence signal using the Olympus Fluorview FV3000 confocal microscope. Data are presented as mean of at least 100 nuclei ± SEM and compared by unpaired t-test; *** P ≤ 0.001.

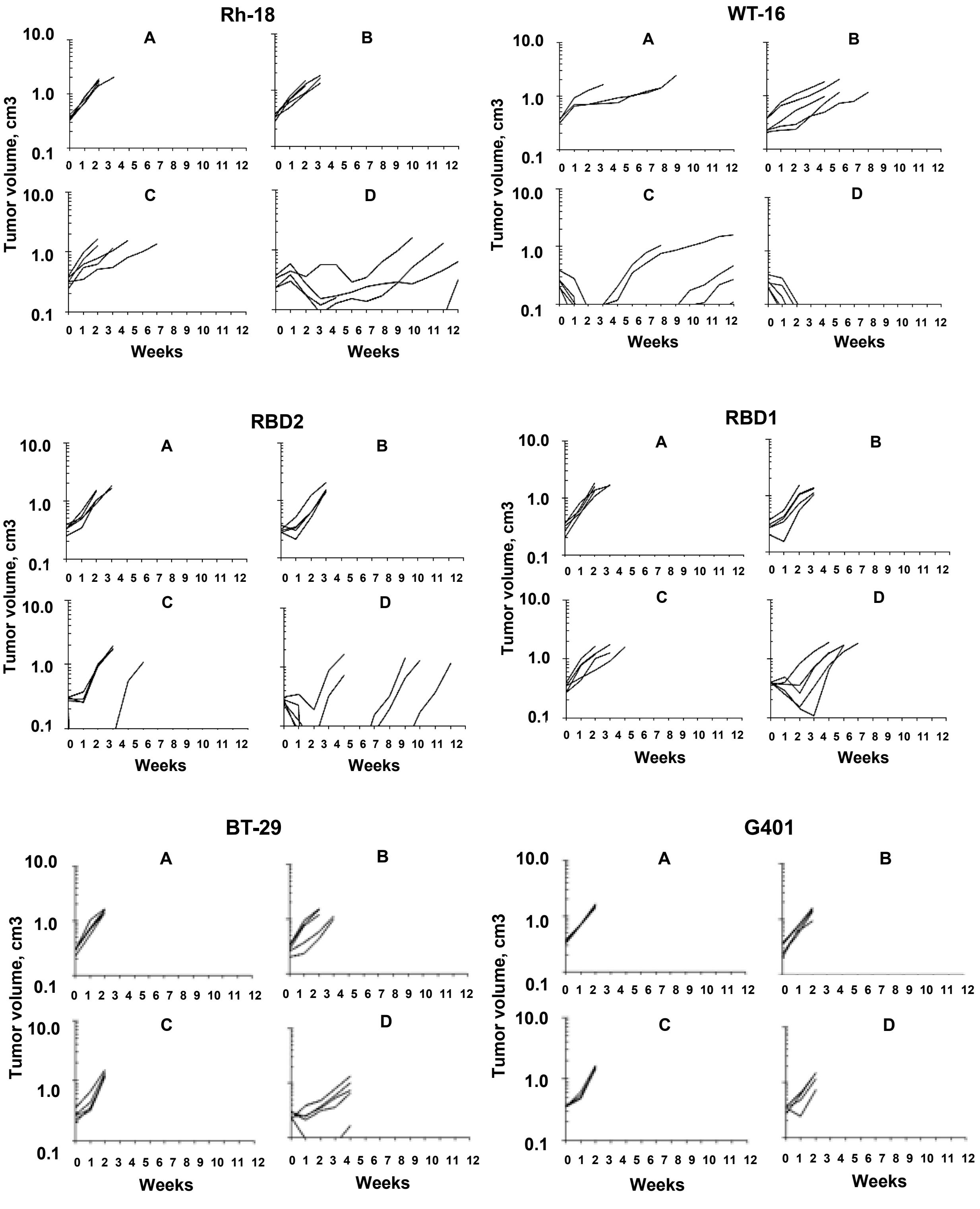

Supplemental Figure S3. Antitumor activity of PEG~TLZ, TMZ, and PEG~TLZ+TMZ in MRT xenografts (in support of Figure 6). Individual treatment group MRT responses for each xenograft model: A-control; B-PEG~TLZ (10 mmol/kg, single dose on day 1); C-TMZ (40 mg/kg daily x 5 on day 4); D-PEG~TLZ+TMZ. Observation period is 12 weeks. Tumors were measured weekly.

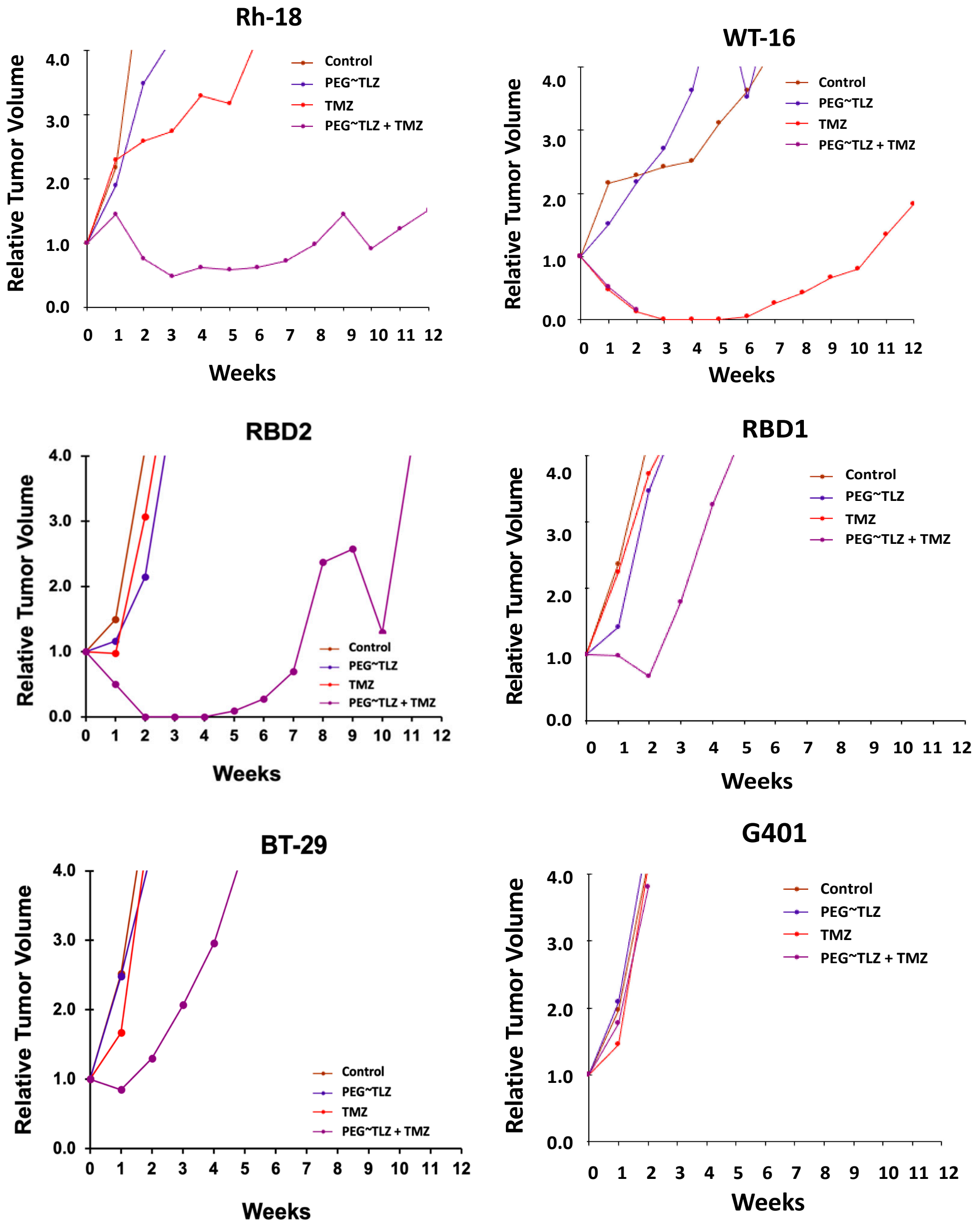

Supplemental Figure S4. Relative tumor volume of MRT xenografts for control, PEG~TLZ, TMZ, and PEG~TLZ+TMZ treatment groups (in support of Figure 6). Each curve represents median relative tumor volume at end of observation period. Brown line-control; Blue line-PEG~TLZ (10 µmol/kg, single dose on day 1); Red line-TMZ (40 mg/kg daily x 5 on day 4); Purple line-PEG~TLZ+TMZ. Observation period of 12 weeks. Tumors were measured weekly.

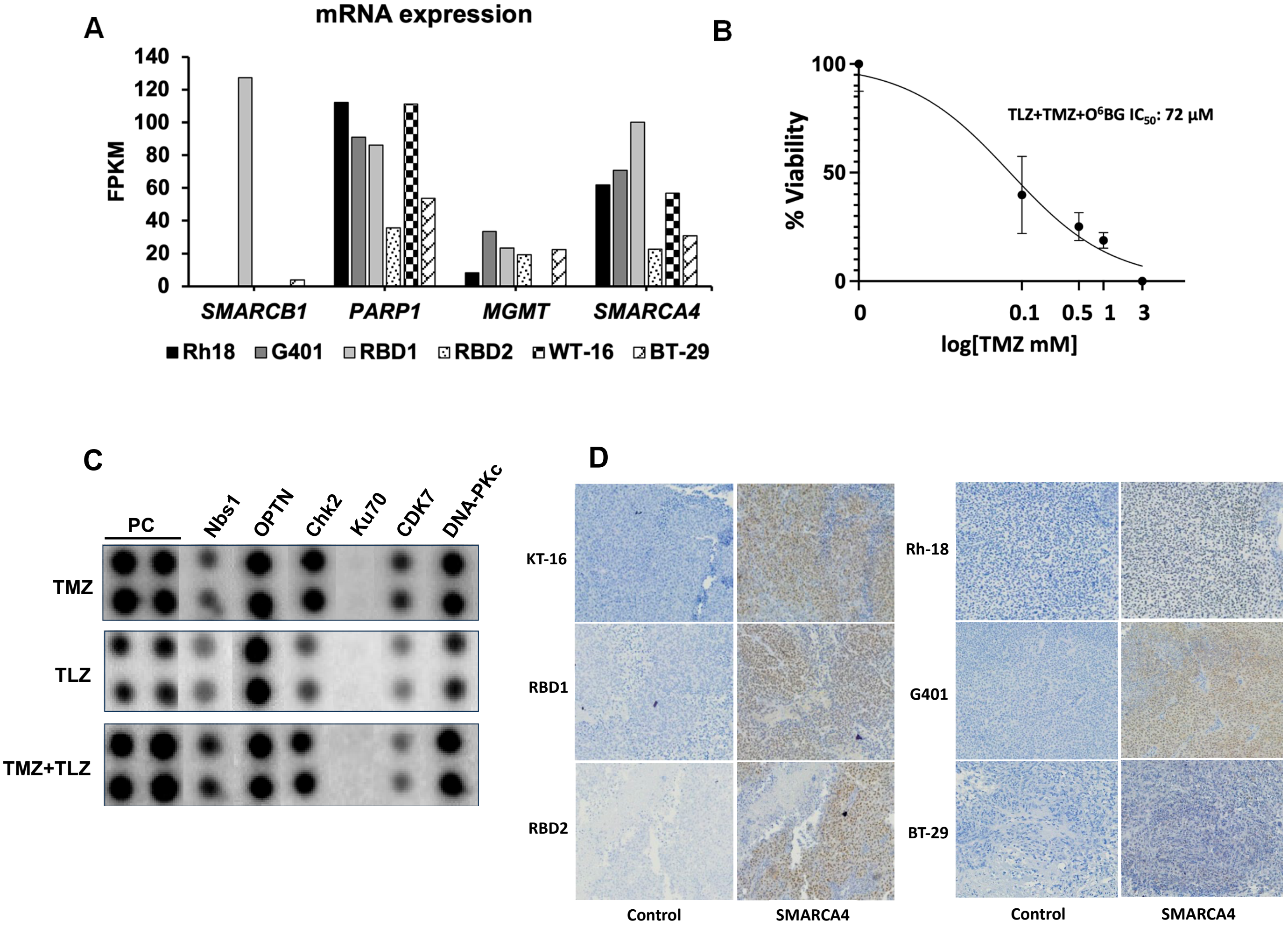

Supplemental Figure S5. DDR in MRT cells and xenografts (in support of Figure 7). ***A,*** mRNA sequencing analysis results. mRNA expression (FPKM) for *SMARCB1, PARP1, MGMT, and SMRACA4* transcripts in MRT xenograft models tested *in vivo*. ***B,*** O^6BG potentiation to TLZ+TMZ in G401$^{SMARCB1+/+}$ cells (see Figure 7D). G401$^{SMARCB1+/+}$ cell potentiation to TLZ+TMZ by MGMT inhibition. Cells were treated with increasing concentrations of TMZ (0-1 mM), TLZ IC_{50} (176 nM), and O^6BG (25 mM). Alamar Blue was added to the plates at 72 h and incubated for 4h, fluorescence was measured at 590 nm (excitation 530 nm) on a Clariostar microplate reader. ***C,*** Representative images of DDR protein expression by antibody array (in support of Figure 7F) in G401$^{SMARCB1+/+}$ cells treated with TMZ (IC_{50}=445 µM), TLZ (IC_{50}=95 nM), and TLZ+TMZ for 72 h. ***D,*** Immunohistochemistry staining for SMARCA4 protein in MRT PDX tumor sections. PC, positive control. DDR, DNA damage response.

A

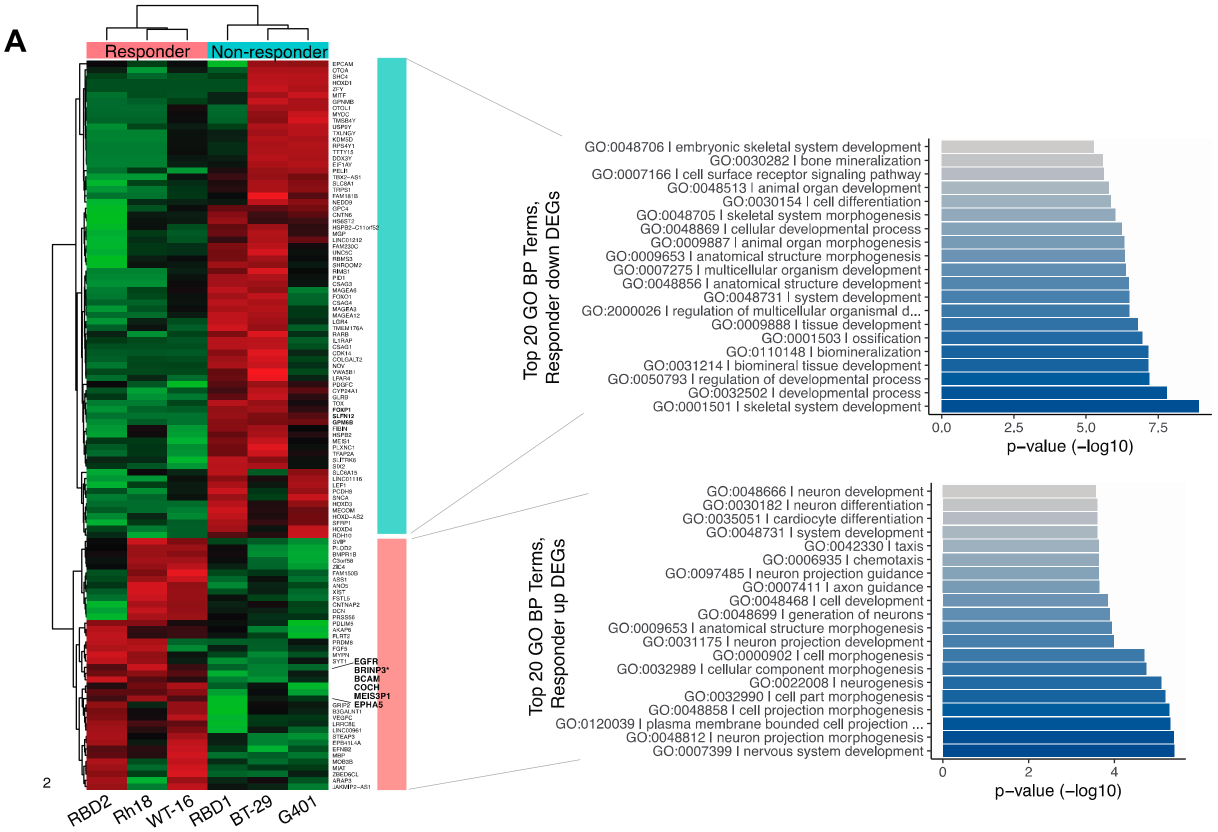

B

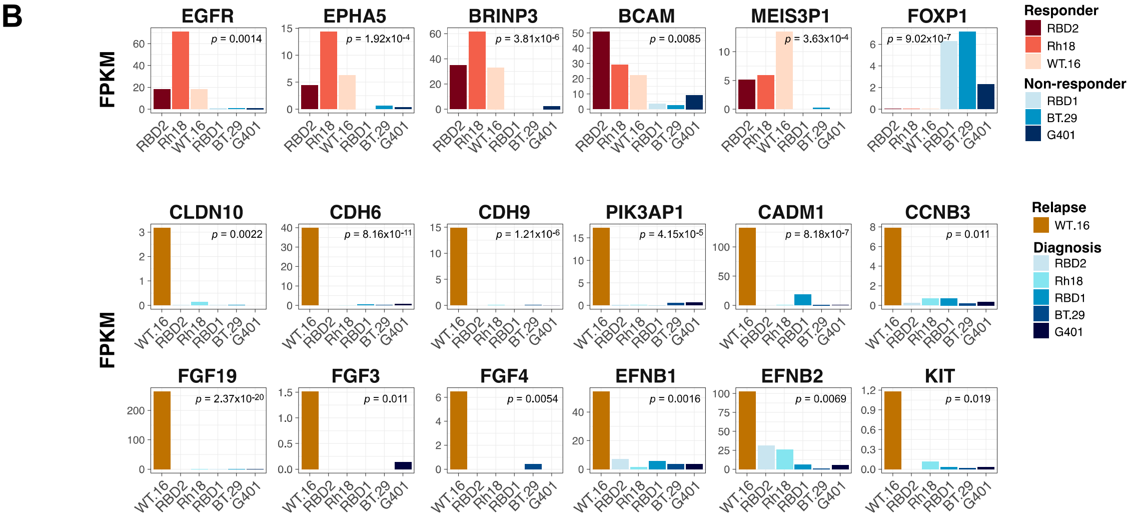

C

PDX	RIN
Rh-18	9.5
WT-16	10.0
RBD2	9.9
RBD1	9.0
BT-29	9.9
G401	9.9

D

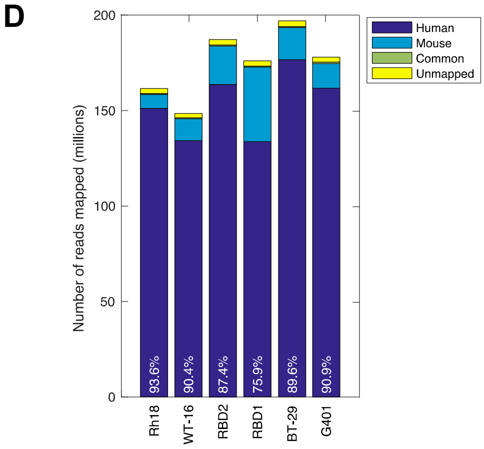

Supplemental Figure S6. Distinctive transcriptomic profile of MRT xenograft tumors responding to PEG~TLZ+TMZ therapy (in support of Tables 3-4). ***A,*** Heatmap showing clustering of DEGs in 'good responders' (RBD2, Rh-18, WT-16) and 'poor responders' (RBD1, BT-29, G401) to PEG~TLZ+TMZ. ***B,*** FPKM values for selected genes upregulated (EGFR, EPHA5, BRINP3, BCAM, MEIS3P1) or downregulated (FOXP1) in the group of 'good responder' xenografts. Highly expressed genes in WT-16 xenograft model only (CLDN10, CDH5, CDH9, PIK3AP1, FGF19, FGF3, FGF4, CADM1, EFNB1, EFNB2, KIT, CCNB3). ***C,*** The RNA integrity (RIN) values for RNAseq of the PDX tumor samples. ***D,*** Proportions of human, mouse, common (to human and mouse) and unmapped reads for the samples used for RNAseq. The percentages provided at the bottom of each bar are percent of human cells over all reads (including unmapped reads). DEG, differentially expressed genes. FPKM, fragments per kilobase of transcript per million mapped reads.

Supplemental Table S1. Confidence intervals of IC_{50} regressions

In support of Figure 1C

TMZ (µM)		TLZ+TMZ (µM)	
G401$^{SMARCB1-/-}$	G401$^{SMARCB1+/+}$	G401$^{SMARCB1-/-}$	G401$^{SMARCB1+/+}$
312.8-496.2	330.7-592.8	62.5-92.4	58.3-104.6

In support of Figure 1D

TLZ (nM)	
G401$^{SMARCB1-/-}$	G401$^{SMARCB1+/+}$
125.4-243.1	56.3-147.1

In support of Supplemental Figure 1A

Cell line	TMZ (µM)	TLZ+TMZ (µM)
BT-16	117.9-192.5	0.8-2.4
BT-12	189.4-282.7	12.2-19.1
Rh-18	388.4-946.6	10.5-96.3

In support of Figure 7D and Supplemental Figure 5B

TLZ+TMZ+O^6BG (µM)	
G401$^{SMARCB1-/-}$	G401$^{SMARCB1+/+}$
25.6-54.4	40.1-115.8

Supplemental Table S2. Clonogenic assay data quantification (in support of Figure 2)

Drugs	Concentration	G401$^{SMARCB1-}$		G401$^{SMARCB1+}$		% decrease	P-value
		Mean	SEM	Mean	SEM		
TMZ	0	161.5	17.6	120.0	21.8	25.7	0.1600
	50 mM	123.0	5.9	116.8	8.0	5.1	0.5082
	100 mM	137.7	9.1	87.0	16.9	36.8	0.0348
	300 mM	77.7	5.2	53.0	2.0	31.8	0.0286
	500 mM	45.7	20.7	19.0	16.1	58.4	0.3695
TLZ	0	162.0	20.4	117.4	28.4	27.5	0.2286
	5 nM	53.3	3.2	42.0	7.5	21.3	0.2702
	15 nM	38.3	1.5	19.7	4.7	48.7	0.0477
	25 nM	19.0	4.9	2.7	1.2	86.0	0.0729
	50 nM	0.0	0.0	0.0	0.0	-	-
TLZ+TMZ	0	161.5	17.6	123.4	19.8	23.6	0.1705
	5 nM+50 mM	53.7	3.2	3.0	0.4	94.4	0.0035
	5 nM+100 mM	19.8	6.3	0.7	0.3	96.6	0.0279
	15 nM+50 mM	21.0	2.6	0.0	0.0	100.0	-
	15 nM+100 mM	2.5	1.3	0.0	0.0	100.0	-
	25 nM+50 mM	0.0	0.0	0.0	0.0	-	-
	25 nM+100 mM	0.0	0.0	0.0	0.0	-	-

SEM, standard error of the mean.
