# Supplementary figures and images for: Synergistic Antitumor Activity of Talazoparib and Temozolomide in Malignant Rhabdoid Tumors
Source: Cancers (Basel). 2024 May 28;16(11):2041. doi: 10.3390/cancers16112041 (PMC11171327; doi:10.3390/cancers16112041)

Figure 1A

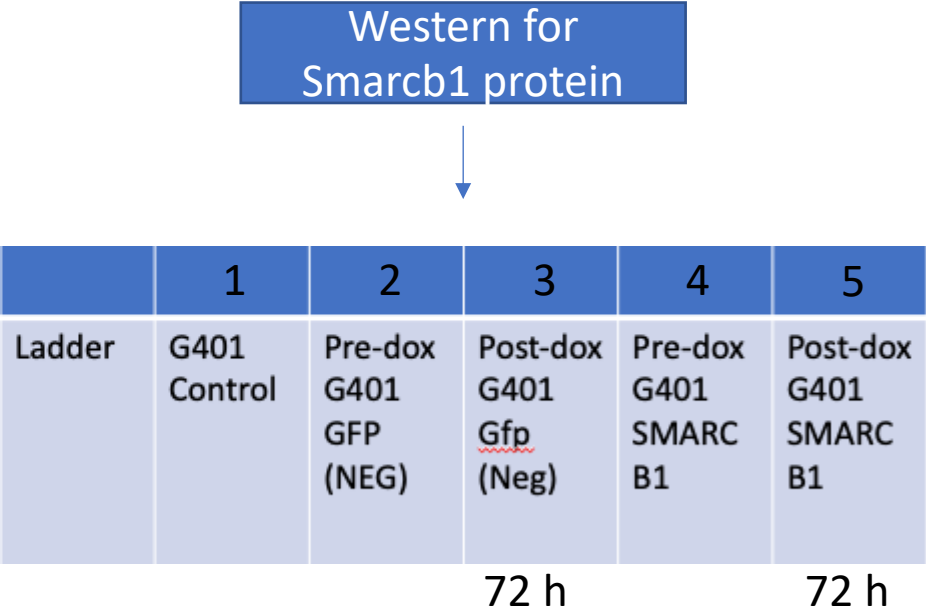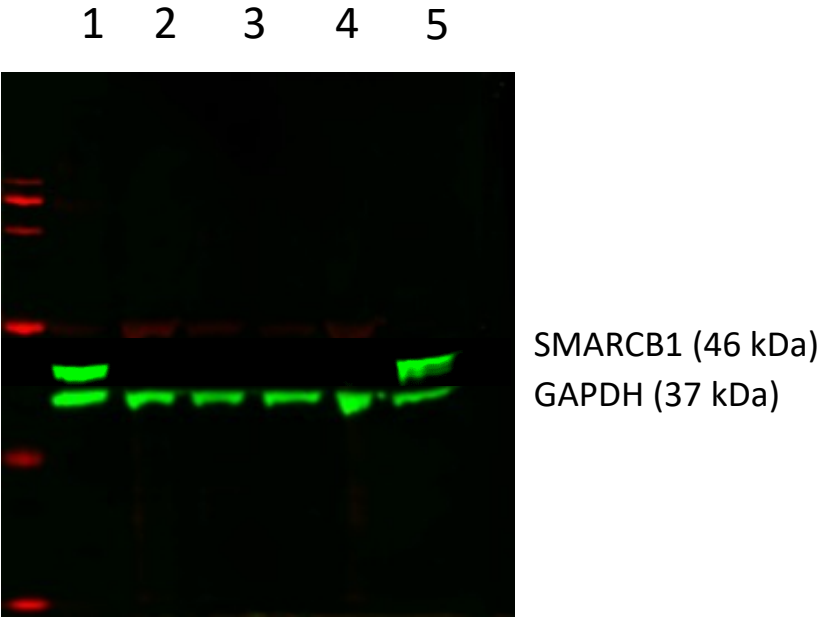

Figure 1B

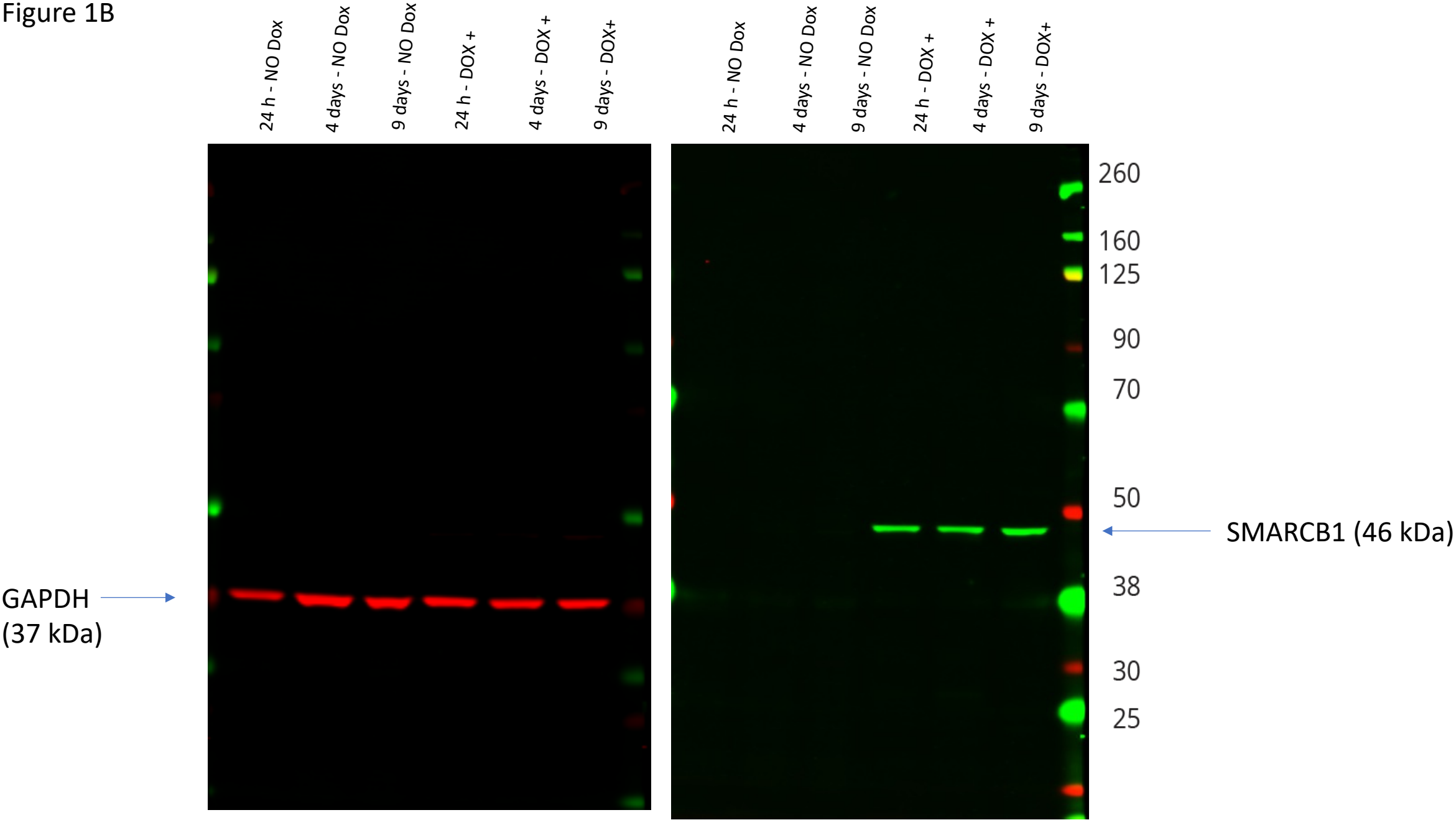

Figure 7A

# Western blot for PARP1, SMARCB1, MGMT, and GAPDH proteins

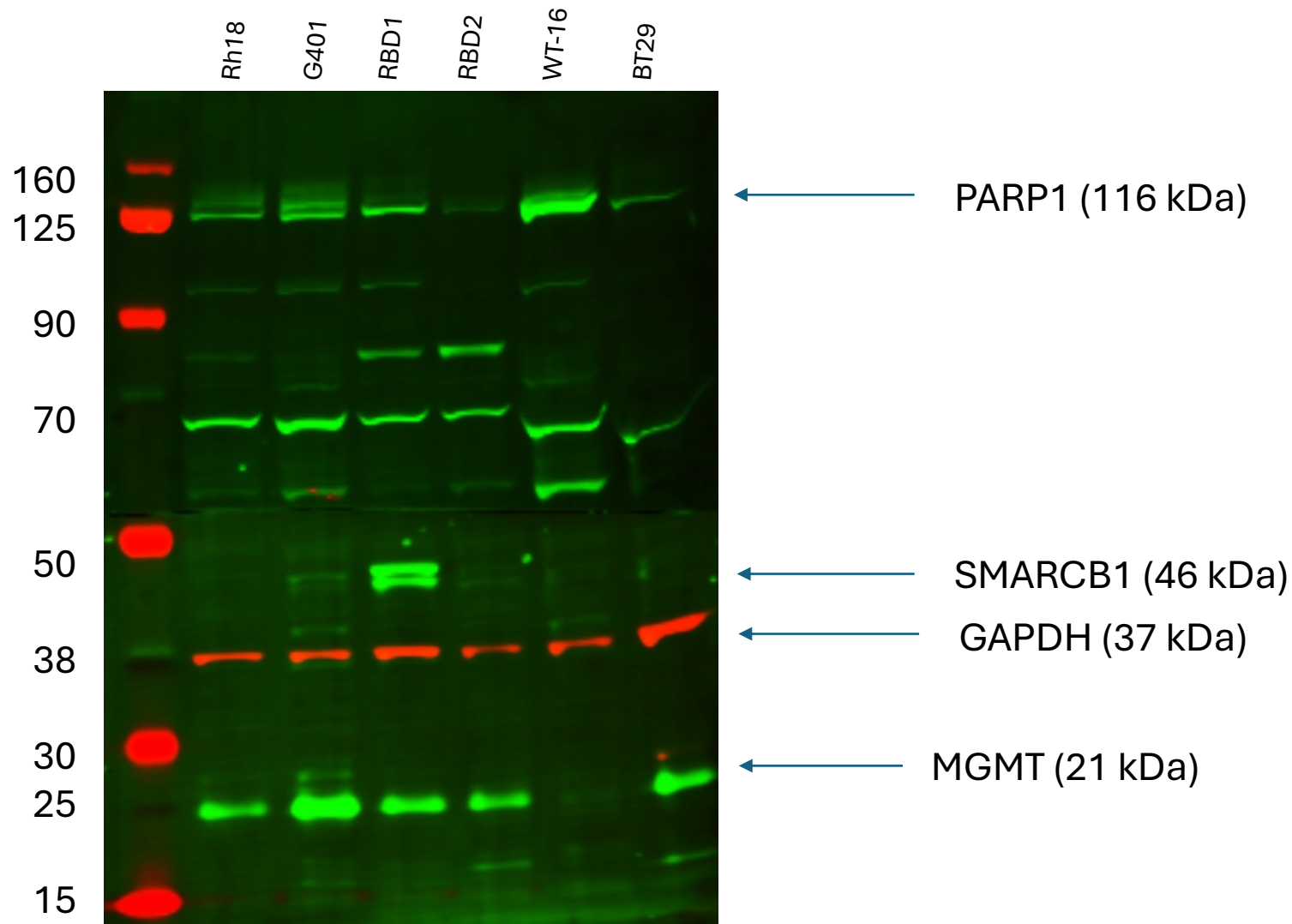

Supplement: Supplementary file 1 [file cancers-16-02041-s001.zip › cancers-3003518-File S1.pdf]
